# ETV2 Overexpression Promotes Efficient Differentiation of Pluripotent Stem Cells to Endothelial Cells

**DOI:** 10.1002/bit.28979

**Published:** 2025-03-25

**Authors:** Yunfeng Ding, Soniya Tamhankar, Feifan Du, Tessa Christopherson, Nate Schlueter, Jenna R. Cohen, Eric V. Shusta, Sean P. Palecek

**Affiliations:** ^1^ Department of Chemical and Biological Engineering University of Wisconsin–Madison Madison Wisconsin USA; ^2^ Department of Neurological Surgery University of Wisconsin–Madison Madison Wisconsin USA

**Keywords:** blood‐brain barrier, differentiation, endothelial cells, ETV2, pluripotent stem cells, transcription factor

## Abstract

Differentiating endothelial cells (ECs) from human pluripotent stem cells (hPSCs) typically takes 2 weeks and requires parameter optimization. Overexpression of cell type‐specific transcription factors in hPSCs has shown efficient differentiation into various cell types. ETV2, a crucial transcription factor for endothelial fate, can be overexpressed in hPSCs to induce rapid and facile EC differentiation (iETV2‐ECs). We developed a two‐stage strategy which involves differentiating inducible *ETV2‐*overexpressing hPSCs in a basal induction medium during stage I and expanding them in an endothelial medium during stage II. By optimizing seeding density and medium composition, we achieved 99% pure CD31+ CD144+ iETV2‐ECs without cell sorting in 5 days. iETV2‐ECs demonstrated in vitro angiogenesis potential, LDL uptake, and cytokine response. Transcriptomic comparisons revealed similar gene expression profiles between iETV2‐ECs and traditionally differentiated ECs. Additionally, iETV2‐ECs responded to Wnt signaling agonist and TGFβ inhibitor to acquire brain EC phenotypes, making them a scalable EC source for applications including blood‐brain barrier modeling.

**Abbreviations**
BBB: blood‐brain barrier
EB: embryoid body
EC: endothelial cell
ESC: embryonic stem cell
hPSC: human pluripotent stem cell
iETV2‐ECS: induced ETV2 ECs
iPSC: induced pluripotent stem cell

## 1 Introduction

Vascular endothelial cells (ECs) form the blood vessel lumen, where they play key roles in the development and maintenance of the circulatory system (Trimm and Red‐Horse 2023). In the past decade, advancements in human pluripotent stem cell (hPSC) technologies have provided a highly scalable source of human endothelial cells, enabling significant progress in modeling human disease, drug testing, and studies of vascular development. Various strategies have been developed to differentiate ECs from hPSCs. Initial strategies formed embryoid bodies (EB) from hPSCs with subsequent isolation of endothelial‐like populations via cell sorting based on the expression of endothelial‐specific cell surface proteins. These methods take 12–15 days on average with approximately 2%–20% of cell population expressing endothelial markers before sorting (Garcia‐Alegria et al. 2021; Goldman et al. 2009; Kim et al. 2007; Levenberg et al. 2002; Liersch et al. 2006). Non‐EB two‐dimensional directed differentiation systems have also been developed in an effort to more efficiently generate ECs from hPSCs. These protocols take 9 to 15 days to generate 10%–60% ECs in differentiated populations (Kane et al. 2010; Lian et al. 2014), and a cell sorting step is generally required to purify hPSC‐derived ECs. In these protocols, differentiating cells are driven into mesoderm progenitors before specification into endothelial lineages. Particular strategies for directing endothelial fates include timed differentiation culture medium supplementation of growth factors such as activin A, BMP4, FGF2, EGF and VEGF, supplementation of hydrocortisone, and inhibition of TGFβ signaling (Costa et al. 2013; Figueiredo et al. 2015; James et al. 2010; Kane et al. 2010; Lian et al. 2014; Orlova et al. 2014; Vodyanik et al. 2010). Efforts have also been made to develop endothelial differentiation protocols more suitable for scaled therapeutic manufacturing, including xenogeneic‐free methods (Luo et al. 2021), highly scalable methods (Lin et al. 2018), and methods offering improved comparability across cell lines (Jahan and McCloskey 2020).

Recently, researchers have developed protocols to derive ECs from hPSCs by directed overexpression of ETS family or SOX family transcription factors. *ETV2*, or ETS variant transcription factor 2, is a critical transcription factor in the regulation of endothelial and hematopoietic development (Koyano‐Nakagawa and Garry 2017). Global *ETV2* knockout has been shown to lead to embryonic lethality at E9.5, with a striking, complete absence of cells from hematopoietic and endothelial lineages (Ferdous et al. 2009). Overexpression of *ETV2* in hPSCs or hPSC‐derived mesoderm progenitors has been shown to drive endothelial cell fate commitment (Luo et al. 2024; Ng et al. 2020; Wang et al. 2020). SOX family transcription factors also play important roles in endothelial development. *SOX7*, *SOX17*, and *SOX18* have been shown to be redundant in function, yet essential for vascular morphogenesis (Lange et al. 2014; Zhou et al. 2015). Recently, overexpression of *SOX17* in hPSCs, in conjunction with FGF2 treatment, has been shown to drive direct reprogramming of hPSCs to endothelial progenitors (Ream et al. 2024). In recent years, several groups have used *ETV2* or *SOX17* to drive endothelial differentiation (Luo et al. 2024; Ng et al. 2020; Ream et al. 2024; Rieck et al. 2024; Wang et al. 2020). These protocols were able to generate approximately 90% CD31+ CD144+ cell populations, presenting a substantial improvement versus EC differentiation protocols using small molecules and growth factors (Luo et al. 2024; Rieck et al. 2024). This exciting progress motivates engineering efforts to further enhance the differentiation process to consistently produce essentially pure (>99%) ECs without the need of cell sorting, thus enabling scalable manufacturing of hPSC‐derived ECs. To this end, we demonstrate that hPSCs, genetically modified to allow the control of *ETV2* expression under an inducible promoter, can generate >99% CD31+ CD144+ endothelial populations in 5 days without sorting, when also using optimized differentiation and expansion media. Transcriptomic, protein, and functional analysis revealed these iETV2‐EC populations have strong expression of endothelial proteins and exhibit key EC functions such as uptake of acetylated LDL and adhesion molecule overexpression upon cytokine stimulation. Moreover, as one potential application, the naïve iETV2‐ECs respond to cues that were previously demonstrated to impart specialized blood‐brain barrier characteristics in mesodermally‐derived ECs.

## 2 Results

### 2.1 Construction of hPSC Lines With Inducible *ETV2* Expression

**Figure 1:**
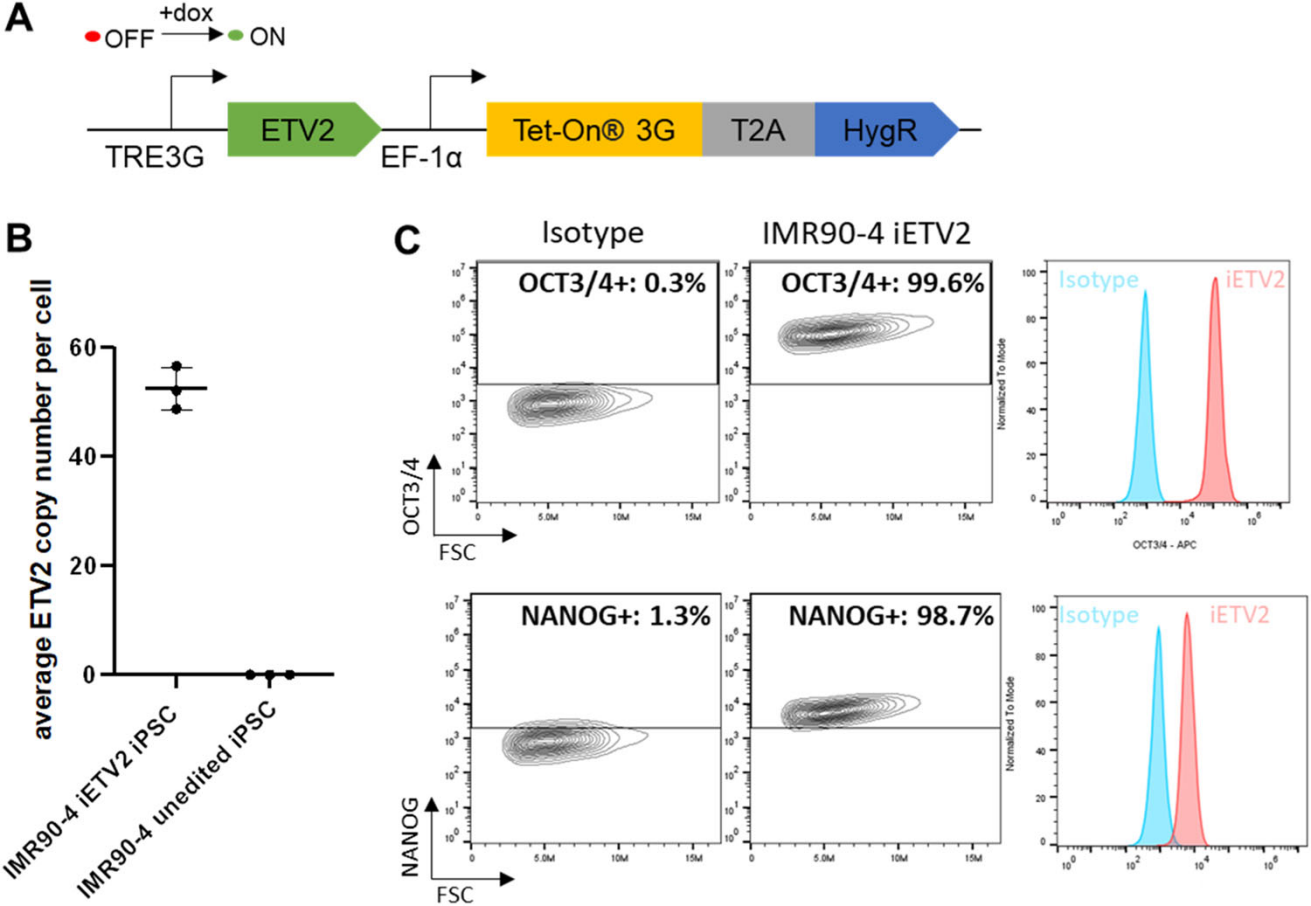
Development of a high copy number, inducible *ETV2* expression IMR90‐4 iETV2 line. (A) A Tet‐on expression system for inducible *ETV2* expression was designed and integrated into IMR90‐4 iPSCs using PiggyBac technology. In this expression construct, *ETV2* expression is initiated by the supplementation of doxycycline in cell culture medium. Expression of the Tet‐On 3G protein is constitutive under the EF‐1α promoter. (B) qPCR of genomic DNA of edited, clonally‐selected, and unedited IMR90‐4 iPSCs indicated an average of 52 genomic copies of iETV2 constructs in the edited clone. *N* = 3 independent genomic DNA isolations from each line. (C) Flow cytometry of IMR90‐4 iETV2 iPSCs indicated that IMR90‐4 iETV2 iPSCs continue to express pluripotency markers OCT3/4 and NANOG.

We created an inducible Tet‐On expression system for *ETV2* (iETV2) and integrated the constructs into the IMR90‐4 iPSC line using the PiggyBac transposase system, where the expression of *ETV2* is doxycycline‐inducible, and the integration of the construct is selectable by hygromycin resistance (Figure 1A). We isolated clones grown from individual edited IMR90‐4 iETV2 iPSCs and performed quantitative PCR (qPCR) on isolated genomic DNA to determine the copy number of *ETV2*. Out of the 9 clones analyzed, we observed a wide distribution of integrated copy numbers from 2 to 52. (Supporting Information S1: Figure S1A). We chose the clone having the highest copy number (52) for detailed characterization to ensure sufficient ETV2 overexpression (Figure 1B). Flow cytometry analyses indicated that edited IMR90‐4 iETV2 iPSCs maintained expression of pluripotency markers OCT3/4 and NANOG (Figure 1C), and the clone exhibited a normal karyotype (Supporting Information S1: Figure S1B). Similarly, we also integrated the iETV2 construct into the H9 embryonic stem cell (ESC) line. We isolated a clone with an iETV2 copy number of 30 (Supporting Information S1: Figure S1C) and validated that the edited line maintained expression of pluripotency marker OCT3/4 (Supporting Information S1: Figure S1D).

### 2.2 Inducible *ETV2* Overexpression Efficiently Drives hPSC Differentiation to CD31 + CD34 + EPCs

**Figure 2:**
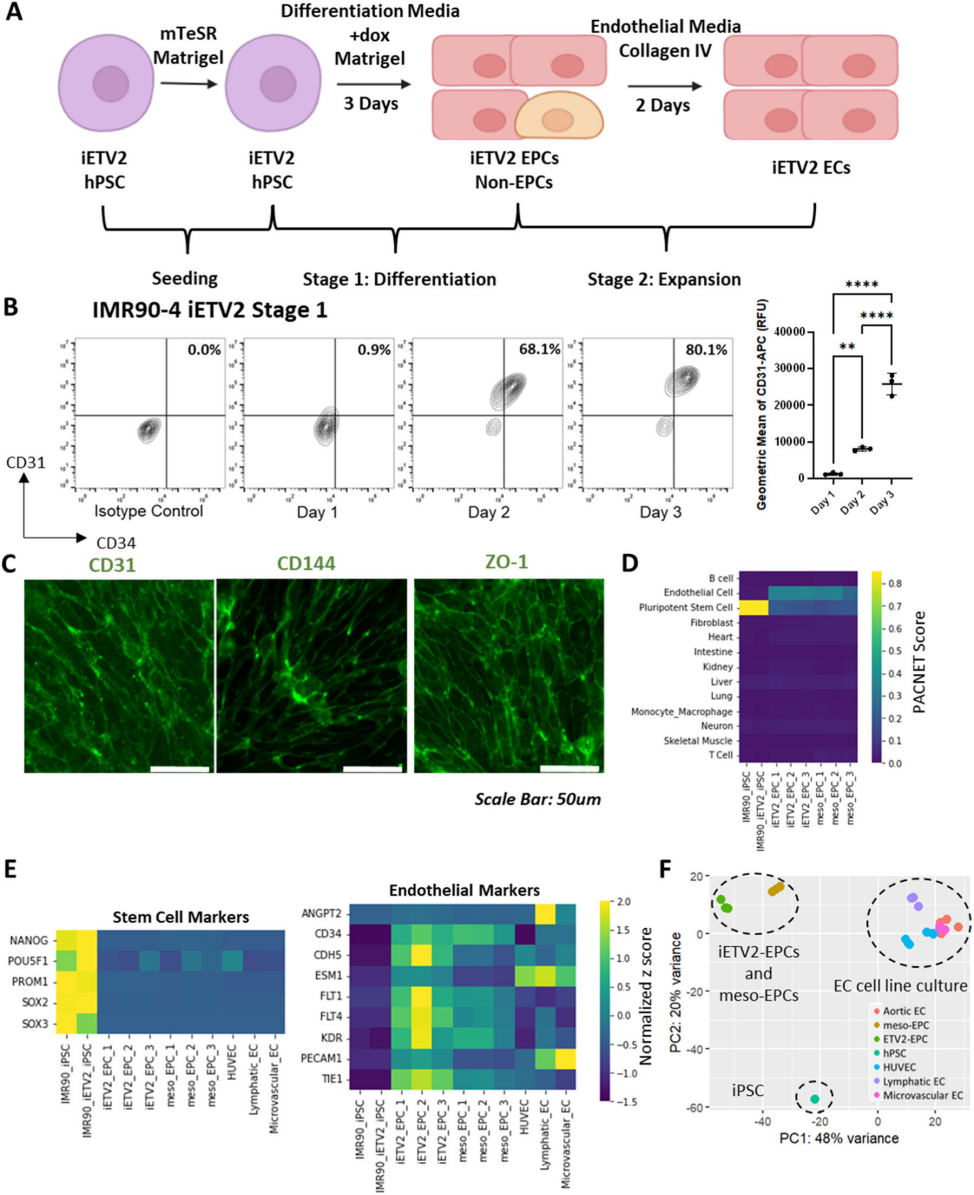
Three days of differentiation by induced *ETV2* overexpression yields iETV2‐EPCs with gene expression similarity to meso‐EPCs. (A) Differentiation and expansion of iETV2‐ECs from hPSCs was carried out in a two‐stage protocol. In stage 1, hPSCs were seeded on Matrigel‐coated plates. 1 µg/mL doxycycline was supplied for three days in a differentiation medium to initiate transient expression of *ETV2* to obtain iETV2‐EPCs. In stage 2, iETV2‐EPCs were seeded on collagen IV in an endothelial medium, yielding high‐purity ETV2‐ECs. (B) Flow cytometry analysis showing the time‐course co‐expression of endothelial progenitor markers CD31 and CD34 after 1 day, 2 days, or 3 days of ETV2 overexpression in mTeSR medium during Stage 1 of the differentiation. Geometric means of CD31 at these time points were quantified. *N* = 3 independent differentiations for each condition quantified. **: *p* < 0.01; ****: *p* < 0.0001 in One‐way ANOVA followed by Tukey's test. (C) Immunofluorescent images showing CD31, CD144, and ZO‐1 expression in Day 3 cells differentiated from IMR90‐4 iETV2 iPSCs. Scale bars are 50 μm. (D) PACNet analysis of transcriptome profiles of iPSC line IMR90‐4, edited iPSC line IMR90‐4 iETV2, iETV2‐EPCs, and meso‐EPCs. EPC populations were MACS‐purified based on CD31 expression before sequencing. (E) Heatmap indicating the expression of a panel of pluripotent stem cell markers (*NANOG, POU5F1, PROM1, SOX2, SOX3*) and a panel of endothelial markers (*ANGPT2, CD34, CDH5, ESM1, FLT1, FLT4, KDR, PECAM1, TEK, TIE1*) in iPSC line IMR90‐4, edited iPSC line IMR90‐4 iETV2, iETV2‐EPCs, meso‐EPCs, HUVECs, primary lymphatic ECs and primary microvascular ECs. HUVEC data is from Grath and Dai 2024. Primary lymphatic and microvascular EC data is from Lim et al. 2019. (F) Principal component analysis of gene expression profiles of IMR90‐4 iPSCs, iETV2‐EPCs, meso‐EPCs, and a variety of cultured EC cell lines, including human aortic EC, lymphatic EC, microvascular EC, and HUVEC.

We designed a two‐stage method to sequentially drive iETV2‐EC differentiation and expansion (Figure 2A). In the differentiation stage, inducible expression of *ETV2* rapidly converts iETV2 hPSCs to endothelial progenitor cells (iETV*2*‐EPCs) during 3 days in differentiation medium that is supplemented with doxycycline. In the expansion stage, iETV2‐EPCs were reseeded onto collagen IV and expanded in endothelial medium to yield iETV2‐ECs. For stage 1, we performed a time‐course study on the effect of inducible *ETV*2 expression on the expression of the endothelial marker CD31 and the endothelial progenitor marker CD34 using mTeSR as the differentiation medium with doxycycline supplementation (Figure 2A). We observed that approximately 80% of cells gained robust expression of CD31 and CD34 in 3 days of differentiation, with increasing expression of CD31 as indicated by increasing geometric mean of CD31 in flow cytometry analyses (Figure 2B). Immunocytochemistry of Day 3 iETV2‐EPCs confirmed junctional expression of endothelial markers CD31, CD144, and ZO‐1 (Figure 2C). We then performed RNA sequencing with Day 3 iETV2‐EPCs to assess the acquisition of an EC transcriptome. First, we confirmed the decrease in the signature of undifferentiated iPSCs and the acquisition of an endothelial signature by PACNet analysis (Figure 2D). We next compared the expression of endothelial markers and pluripotency markers for IMR90‐4 iETV2‐EPCs with IMR90‐4 EPCs that were instead derived through mesoderm progenitors (meso‐EPCs) via modulating Wnt signaling using CHIR99021 as previously described (Lian et al. 2014). As expected, both iETV2‐EPCs and meso‐EPCs had reduced expression of markers for undifferentiated iPSCs and acquired expression of endothelial genes. Expression of EC markers is comparable to HUVECs (Grath and Dai 2024), primary lymphatic ECs, and primary microvascular ECs (Lim et al. 2019) (Figure 2E). Moreover, we assessed the expression of a list of 125 previously published pan‐endothelial genes (Schupp et al. 2021) and found that iETV2‐EPCs had similar expression levels of these pan‐endothelial genes to meso‐EPCs, with a correlation score of 0.795 (Supporting Information S1: Figure S2). A principal component analysis on transcriptomic profiles of undifferentiated iPSCs, iETV2‐EPCs, meso‐EPCs, and various primary, cultured EC sources indicated that transcriptomic profiles of iETV2‐EPCs were closest to those of meso‐EPCs along the first principal component and were most similar to cultured ECs along the second principal component (Figure 2F). We performed a differential gene expression analysis between ETV2‐EPCs and meso‐EPCs using DESeq2 and found that 18% of genes (3682 genes) were significantly upregulated in iETV2‐ECs, while 18% of genes (3793 genes) were upregulated in meso‐ECs (Supporting Information S1: Figure S3A). We also performed gene ontology (GO) analysis on differentially expressed genes (Supporting Information S1: Figures S3B, S3C). Notably, several GO terms associated with nervous system development were enriched in ETV2‐EPCs (Supporting Information S1: Figure S3B). This enrichment may result from meso‐EPCs transiting through mesoderm germ layer during directed differentiation, but ETV2‐EPCs not following this developmental trajectory. On the other hand, several key Wnt signaling genes (*LEF1, FOXF1, CDH13, GPC3*) were upregulated in meso‐EPCs (Supporting Information S1: Figure S3A). Considering that Wnt agonist CHIR99021 was used during mesoderm specification, enhanced expression of Wnt signaling genes in meso‐EPC is consistent with induction of canonical Wnt signaling. We also observed collagen genes (*COL1A1*, *COL3A1*) to be more highly expressed in meso‐EPCs than in ETV2‐EPCs (Supporting Information S1: Figure S3A). Overall, we found that inducible *ETV2* expression drives rapid acquisition of EPC markers CD31 and CD34 in just 3 days, as a relatively pure population. The resultant iETV2‐EPC population gained endothelial gene expression and exhibited high transcriptomic similarity to previously reported meso‐EPCs.

### 2.3 Optimization of Stage 1 Differentiation Medium and Cell Seeding Density During Induced *ETV2* Overexpression Generates Highly Pure EC Populations

**Figure 3:**
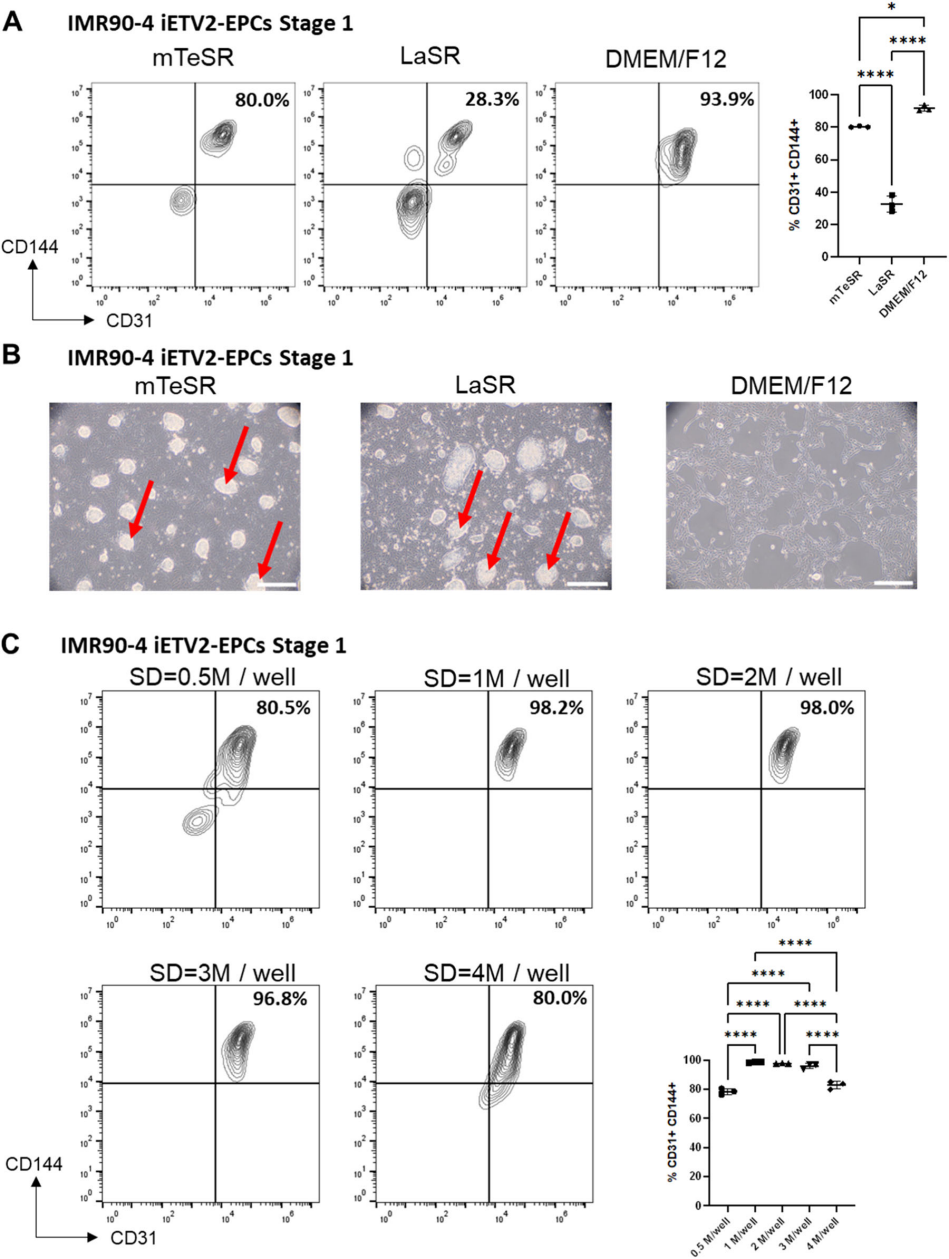
Using basal medium DMEM/F12 during differentiation stage 1 yields high purity CD31+ CD144+ populations. (A) Flow cytometry analysis of CD144 and CD31 expression of Day 3 IMR90‐4 iETV2‐EPCs differentiated in mTeSR, LaSR, or DMEM/F12 during Stage 1. Seeding density was 1 million cells/well in a six‐well plate. *N* = 3 independently differentiated biological replicates were quantified. *: *p* < 0.05; ****: *p* < 0.0001 in one‐way ANOVA followed by Tukey's test. (B) Bright field microscopy images on Day 3 cells differentiated in mTeSR, LaSR or DMEM/F12 during Stage 1. Arrows point to examples of larger non‐EPC cell aggregates. Scale bars are 100 µm. (C) Flow cytometry analysis of CD144 and CD31 expression of Day 3 cells differentiated in DMEM/F12 with varying seeding density of IMR90‐4 iETV2 iPSCs on Day ‐1. *N* = 3 independently differentiated biological replicates were quantified. ****: *p* < 0.0001 in one‐way ANOVA followed by Tukey's test.

As mentioned earlier, most current endothelial differentiation protocols require magnetic or fluorescence‐activated cell sorting to purify endothelial populations, a labor‐intensive and time‐consuming step that makes upscaling of EC differentiation batches challenging (Costa et al. 2013; Garcia‐Alegria et al. 2021; Goldman et al. 2009; Lian et al. 2014). To explore whether the medium used during the differentiation stage could be manipulated to yield high‐purity iETV2‐EPCs, we tested three different differentiation medium formulations: mTeSR (as used in Figure 2), a medium commonly used for self‐renewal and maintenance of hPSCs (Lannon et al. 2008); LaSR, a medium commonly used for mesoderm differentiation (Bao et al. 2015); and DMEM/F12, a basal medium lacking growth factors or small molecule signaling components (Dulbecco and Freeman 1959; Ham 1965). By flow cytometry, we found that DMEM/F12 consistently generated more than 90% CD31+CD144+ EPCs with few, if any, CD31‐CD144‐ non‐EPCs. By contrast, mTeSR generated 80% CD31+CD144+ EPCs and LaSR generated only around 30% CD31+CD144+ EPCs, with many CD31‐CD144‐ non‐EPCs (Figure 3A). By co‐staining cells with anti‐CD31 and anti‐CD34 antibodies, we found that regardless of the differentiation medium used in Stage 1, virtually all CD31+ cells also expressed CD34, with DMEM/F12 generating the purest dual positive population (Supporting Information S1: Figure S4A). Concordant with these purities, visual inspection of Day 3 differentiated cells by brightfield microscopy revealed colony‐like aggregates of non‐EPC‐like cells in mTeSR and LaSR conditions, while the cobblestone‐like morphology signature to ECs was present in DMEM/F12 conditions throughout the culture plate (Figure 3B). Using DMEM/F12 as the base medium for differentiation during Stage I, we then tested the effect of different initial iPSC seeding densities on EPC purity. We found that seeding 1 million to 3 million IMR90‐4 iETV2 iPSCs per well in a six‐well plate (1.04 × 10^5^ to 3.13 × 10^5^ cells/cm^2^), seeding densities that generate a nearly confluent monolayer after 24 h, consistently generated nearly pure EPC populations containing more than 95% CD31+ CD144+ EPCs (Figure 3C). We also replicated the Stage 1 differentiation using DMEM/F12 and 2 million/well seeding density in the H9 iETV2 ESC line and observed over 95% CD31+ CD144+ EPC purity (Supporting Information S1: Figure S4B), indicating the generalizability of the differentiation to an hESC line. One factor of particular interest is insulin, as it is widely used in medium for maintaining the undifferentiated state (Lannon et al. 2008; Ludwig and A Thomson 2007) and for driving cell fate decisions in directed differentiation protocols (Duan et al. 2018; Freund et al. 2008; Lian et al. 2013). We explored the effect of the addition of insulin in the DMEM/F12 differentiation medium and observed a significant decrease in the purity of CD31+ CD144+ EPCs, suggesting that insulin supplementation is detrimental to ETV2‐mediated forward programming (Supporting Information S1: Figure S4C). Overall, with optimized medium choice and seeding density during the Stage 1 differentiation phase, we were able to efficiently achieve a highly pure iETV2‐EPC population without the need for cell sorting.

### 2.4 Optimization of Stage 2 Expansion Medium Allows for Maintenance of EC Identity and Increased Yield

**Figure 4:**
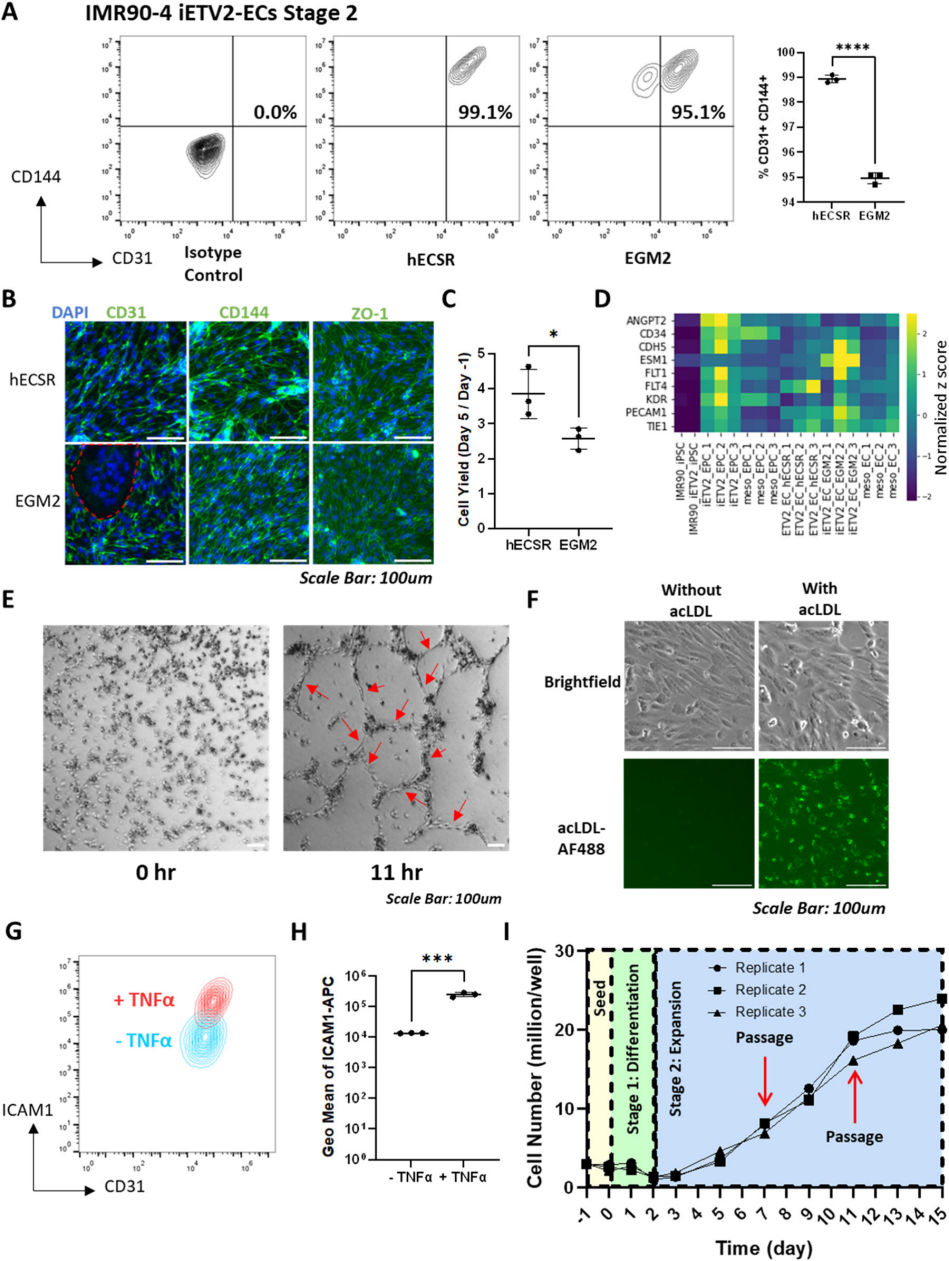
Using hECSR medium during stage 2 generates functional iETV2‐ECs with high purity and improved yield. (A) Flow cytometry analysis of CD144 and CD31 expression on day 5 IMR90‐4 iETV2‐ECs expanded in hECSR or EGM2 for 3 days during Stage 2. *N* = 3 independently differentiated biological replicates were quantified. ****: *p* < 0.0001 in Student's *t*‐test. (B) Immunofluorescent images showing CD31, CD144, and ZO‐1 expression in Day 5 iETV2‐ECs differentiated from the IMR90‐4 iETV2 cell line using DMEM/F12 during Stage 1 differentiation and hECSR or EGM2 during Stage 2 expansion. Areas that are DAPI+ but CD31‐ were circled in red dashed lines. Scale bars are 100 μm. (C) Yield of IMR90‐4 iETV2 cells on Day 5 expanded using either hECSR or EGM2 as Stage 2 medium. Yield is defined as the number of iETV2‐ECs on Day 5 divided by the number of iPSCs seeded on Day ‐1. *N* = 3 independently differentiated biological replicates were quantified. *: *p* < 0.05 in Student's t‐test. (D) Heatmap indicating the expression of a panel of endothelial markers (*ANGPT2, CD34, CDH5, ESM1, FLT1, FLT4, KDR, PECAM1, TEK, TIE1*) of IMR90 iPSCs, iETV2‐EPCs and meso‐EPCs, iETV2‐ECs and meso‐ECs. (E) Cord formation assay for iETV2‐ECs at 0 h and 11 h after medium change to angiogenic, low serum growth supplement. Red arrows indicate nascent cords at 11 h. Additional time points and EC controls can be found in Supporting Information S1: Figure S9. (F) Brightfield and immunofluorescence images of Day 5 iETV2‐ECs differentiated from the IMR90‐4 iETV2 cell line using DMEM/F12 during Stage 1 and hECSR or EGM2 during Stage 2. Images were taken with or without 24 h incubation with Alexa Fluor 488‐labeled acetylated LDL (acLDL‐AF488). Scale bars are 100 μm. (G) Flow cytometry analysis of ICAM1 expression after 24 h of incubation with or without TNF‐α in Day 5 iETV2‐ECs differentiated from the IMR90‐4 iETV2 cell line using DMEM/F12 during Stage 1 and hECSR or EGM2 during Stage 2. Red: with TNF‐α. Blue: Without TNF‐α. (H) Geometric mean of ICAM1 expression on flow cytometry analysis performed in (G). *N* = 3 independently differentiated biological replicates were quantified. ***: *p* < 0.001 in Student's *t*‐test. (I) Growth curve quantifying cells numbers during the differentiation process in three independent differentiations of IMR90‐4 iETV2 iPSCs using DMEM/F12 during Stage 1 and hECSR during Stage 2. *N* = 3 independently differentiated biological replicates are shown. Time points where confluent iETV2‐ECs were passaged are indicated by red arrows.

Although we consistently observed more than 95% pure CD31+ CD144+ EPC populations with DMEM/F12 medium at the optimal sending density at Day ‐1, one caveat of using a basal medium without serum or growth factors is that the total cell numbers did not increase during Stage 1 (Figure 4I). We thus tested two different common endothelial growth media during Stage 2 to accommodate EC expansion: hECSR, a serum‐free formulation used for rapid proliferation of ECs (Nishihara et al. 2020), and EGM2, a serum‐containing formulation commonly used for culturing primary endothelial cells (Cheung 2007). During stage 2, the cells were re‐seeded on collagen IV at 30,000 cells/cm^2^. By flow cytometry, we found that using hECSR medium during the 2 days of Stage 2 expansion generated 99% CD31+ CD144+ ECs, while cells cultured in EGM2 contained a 95% CD31+ CD144+ population, along with a clear reproducible population of around 3% CD31‐ cells (Figure 4A). Using the optimized, initial hPSC seeding density, Stage 1 DMEM/F12 and Stage 2 hECSR or EGM2 medium, we next evaluated general EC phenotypes in iETV2‐ECs. Immunocytochemistry of Day 5 iETV2‐ECs confirmed junctional expression of CD31, CD144, and ZO‐1 in iETV2‐ECs expanded in either medium, similar to meso‐ECs and HUVECs (Figure 4B, Supporting Information S1: Figure S5A). Immunocytochemistry also confirmed the existence of CD31‐ clusters of cells in EGM2. (Figure 4B). Compared to EGM2, hECSR also generated a higher cell yield on Day 5 of about 4 iETV2‐ECs per input iPSC (Figure 4C). We also replicated the Stage 2 hECSR expansion with the H9 iETV2 ESC line and observed a 98% CD31+ CD144+ purity (Supporting Information S1: Figure S6), indicating the generalizability to another hESC lines. Finally, to globally compare cells cultured in the two different expansion media during Stage 2, we performed RNA sequencing on sorted CD31+ CD144+ Day 5 iETV2‐ECs. We found that cells cultured in both endothelial media expressed comparable levels of endothelial markers (Figure 4D). When assessing a wide panel of 125 pan‐endothelial genes (Schupp et al. 2021), we found that further expanding iETV2‐EPCs to iETV2‐ECs in hECSR did not lead to significant changes in endothelial gene expression (Supporting Information S1: Figure S7A). Comparing iETV2‐ECs expanded in EGM2 and hECSR, endothelial gene expression was also comparable (Supporting Information S1: Figure S7B). Taken together, given the purer CD31+ CD144+ EC populations resulting from hECSR medium expansion, we moved forward with hECSR as the optimized medium for Stage 2 expansion. Recently, a differentiation protocol has been developed to specify iPSC‐derived ECs into venous lineage (Pan et al. 2024). To assess whether iETV2‐ECs and meso‐ECs have arterial or venous lineage transcriptional signatures, we analyzed the expression of panels of arterial and venous genes and found that neither EC model exhibited a clear signature of arterial or venous lineage specification (Supporting Information S1: Figure S8). Next, to explore angiogenic function of iETV2‐ECs, we performed in vitro endothelial cord formation assays with hECSR‐expanded ECs. We found that Day 5 iETV2‐ECs cultured in hECSR formed nascent cords beginning about 4 h after seeding, with a stable network of cords formed at 11 h (Figure 4E, Supporting Information S1: Figure S9). Meso‐ECs and HUVECs also formed cords in this assay, albeit at differing temporal profiles, with iETV2‐ECs forming cords at rates intermediate to HUVECs and meso‐ECs (Supporting Information S1: Figures S9, S10A). iETV2‐ECs networks also formed the same number of total nodes as meso‐ECs after 11 h, with shorter total cord length than observed with HUVECs (Supporting Information S1: Figures S10A, S10B). Finally, cords formed by the iETV2‐ECs were of intermediate thickness compared with meso‐ECs and HUVECs (Supporting Information S1: Figures S9, S10C). Thus, all EC sources exhibit endothelial network formation in vitro, but with differences in timing and architecture. We further characterized endothelial function of these cells by confirming that iETV2‐ECs, similar to meso‐ECs and HUVECs, internalized acetylated LDL (Figure 4F, Supporting Information S1: Figure S5C) and demonstrated that iETV2‐ECs, similar to meso‐ECs and HUVECs, expressed increased amounts of adhesion molecule ICAM1 upon TNF‐α cytokine stimulation (Figure 4G,H, Supporting Information S1: Figure S5B). We continued culturing iETV2‐ECs for 10 additional days and two passages and quantified the resultant number of ECs. While cell numbers decreased during Stage 1 in basal medium without serum or growth factor supplementation, in Stage 2, cells proliferated in hECSR medium, with a doubling time of approximately 2 days. This EC expansion continued for three doublings before proliferation slowed and by Day 15, an average of 21.5 iETV2‐ECs had been generated per input iPSC (Figure 4I).

### 2.5 ETV2‐ECs Acquire Brain EC Phenotypes Upon Activation of Canonical Wnt Signaling and Inhibition of TGFβ Signaling

ECs in the brain gain special characteristics to form the blood‐brain barrier compared to peripheral counterparts. For example, they exhibit high expression of tight junction proteins (e.g., claudin‐5, occludin), efflux transporters (e.g., p‐glycoprotein, breast cancer resistance protein), and specialized nutrient transporters (e.g., GLUT‐1, MFSD2A). Functionally, they form a tight barrier between the circulatory bloodstream and the central nervous system (CNS), safeguarding CNS homeostasis by regulating the transport of small molecules, proteins, and cells (Daneman and Prat 2015). Previously, it has been shown that activation of canonical Wnt signaling and the inhibition of TGFβ signaling can drive the acquisition of some brain EC phenotypes by hPSC‐derived ECs differentiated through meso‐EPCs (Gastfriend et al. 2021; Roudnicky et al. 2020). In particular, activation of canonical Wnt signaling in hPSC‐derived ECs has been shown to drive the acquisition of some canonical BBB phenotypes, including expression of glucose transporter (GLUT‐1), increased tight junction protein claudin‐5 and decreased permeability (Gastfriend et al. 2021). Inhibition of TGF‐β signaling in hPSC‐derived ECs has been shown to increase claudin‐5 expression and decrease permeability (Roudnicky et al. 2020).

**Figure 5:**
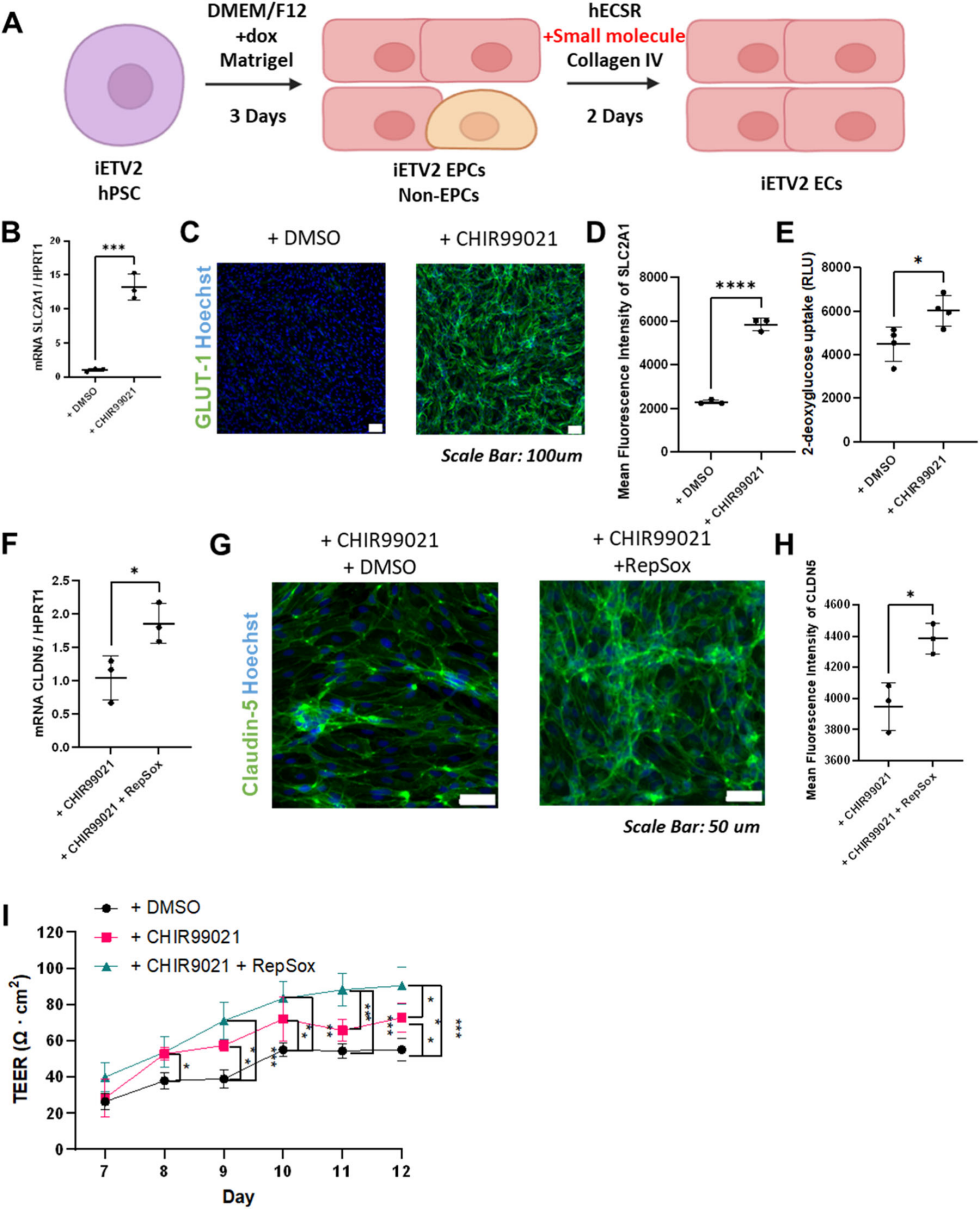
IMR90‐4 iETV2‐ECs respond to known small molecule inducers of BBB properties. (A) Schematic of small molecule induction of ETV2‐ECs. During Stage 2, IMR90‐4 iETV2‐EPCs were seeded in hECSR medium with small molecule inducers CHIR99021 or RepSox on collagen IV matrix for two days before analysis. (B) RT‐qPCR quantifying *SLC2A1* transcript, normalized to housekeeping gene *HPRT1*, on Day 5 for IMR90‐4 iETV2‐ECs treated with Wnt agonist CHIR99021 or DMSO control. *N* = 3 independently treated biological replicates were quantified. ***: *p* < 0.001 using Student's *t*‐test. (C) Immunofluorescent images showing GLUT‐1 expression in Day 5 iETV2‐ECs treated with Wnt agonist CHIR99021 or DMSO control. Scale bars are 100 μm. (D) Quantification of mean GLUT‐1 fluorescence intensity in immunofluorescent images of Day 5 IMR90‐4 iETV2‐ECs treated with Wnt agonist CHIR99021 or DMSO control. *N* = 3 independently treated biological replicates were quantified. ****: *p* < 0.0001 in Student's *t*‐test. (E) 2‐deoxyglucose uptake by day 5 IMR90‐4 iETV2‐ECs treated with Wnt agonist CHIR99021 or DMSO control, as quantified by luciferase activity (see materials and methods for details). *N* = 3 independently differentiated and treated biological replicates for each condition were quantified. *: *p* < 0.05 in Student's *t*‐test. (F) RT‐qPCR quantifying *CLDN5* transcripts, normalized to housekeeping gene *HPRT1*, on Day 5 in IMR90‐4 iETV2‐ECs treated with Wnt agonist CHIR99021 and DMSO control, or Wnt agonist CHIR99021 and TGFβ inhibitor RepSox. *N* = 3 independently differentiated and treated biological replicates for each condition were quantified. *: *p* < 0.05 using Student's *t*‐test. (G) Immunofluorescent images showing Claudin‐5 expression in Day 5 IMR90‐4 iETV2‐ECs treated with Wnt agonist CHIR99021 and DMSO control, or Wnt agonist CHIR99021 and TGFβ inhibitor RepSox. Scale bars are 100 μm. (H) Quantification of mean fluorescence intensity of Claudin‐5 in immunofluorescent images of Day 5 IMR90‐4 iETV2‐ECs treated with Wnt agonist CHIR99021 and DMSO control, or Wnt agonist CHIR99021 and TGFβ inhibitor RepSox. *N* = 3 independently differentiated and treated biological replicates were quantified. *: *p* < 0.05 in Student's *t*‐test. (I) Transendothelial electrical resistance (TEER) of IMR90‐4 iETV2‐ECs treated with or without the indicated small molecules. The *x*‐axis indicates the number of days after initiation of differentiation, and TEER was first measured on Day 7. Points represent the average of replicate wells from four independent differentiations of the IMR90‐4 iETV2 iPSC line, *: *p* < 0.05; **: *p* < 0.01; ***:*p* < 0.001; ****:*p* < 0.0001 in two‐way ANOVA followed by Tukey's test.

We aimed to examine whether modulation of these brain‐EC relevant signaling pathways in iETV2‐ECs would have a similar effect in inducing BBB properties. IMR90‐4 iETV2‐ECs were differentiated and expanded as described above, except during Stage 2 we supplemented the hECSR medium with CHIR99021 (Wnt agonist), CHIR99021 and RepSox (TGFβ inhibitor), or DMSO (control) for 2 days (Figure 5A). By evaluating gene expression using RT‐qPCR, we found that CHIR99021 treatment induced an increase in the *SLC2A1* glucose transporter (GLUT‐1) transcript 13.2‐fold (Figure 5B). Using immunocytochemistry, we also observed a robust 2.5‐fold increase in the expression of GLUT‐1 protein (Figures 5C,D). Similarly, meso‐ECs also responded to CHIR99021 treatment to gain GLUT‐1 expression as previously reported (Gastfriend et al. 2021) (Supporting Information S1: Figure S11). To evaluate glucose uptake capacity after CHIR99021 treatment, we measured 2‐deoxyglucose uptake and found that CHIR99021‐treated iETV2‐ECs had 1.3‐fold elevated glucose uptake capabilities compared to the DMSO control (Figure 5E). We then treated CHIR99021‐induced iETV2‐EPCs with the TGF‐β inhibitor RepSox. Following RepSox treatment, we observed a 1.9‐fold increase in the *CLDN5* transcript (Figure 5F) and immunocytochemistry also showed an increase in CLDN5 protein expression (Figures 5G,H). We then quantified barrier permeability properties of iETV2‐ECs by measuring the trans‐endothelial electrical resistance (TEER). We found that iETV2‐ECs treated with CHIR99021 displayed elevated TEER and additional treatment with RepSox further elevated TEER (Figure 5I), observations previously reported on meso‐EPC‐derived ECs (Gastfriend et al. 2021; Roudnicky et al. 2020). Collectively, these results demonstrated that iETV2‐ECs respond to previously identified small molecule pathway modulators to gain brain‐EC‐enriched gene expression and function.

## 3 Discussion

Direct overexpression of cell‐type specific transcription factors in hPSCs has proven to be an efficient strategy for the generation of somatic cell types (Pawlowski et al. 2017; Tomaz et al. 2022). ETV2 has been identified to be essential for endothelial and hematopoietic lineage specification (Koyano‐Nakagawa and Garry 2017). Recently, *ETV2* overexpression or delivery of modified *ETV2* mRNA has been shown to drive differentiation or trans‐differentiation to endothelial cells (Lindgren et al. 2015; Luo et al. 2024; Morita et al. 2015; Ng et al. 2020; Rieck et al. 2024; Wang et al. 2020). Such progress prompted us to attempt to optimize the *ETV2*‐driven differentiation to consistently generate pure (>99%) CD31+ CD144+ ECs without cell sorting. We developed a two‐stage differentiation system. During stage 1, ETV2 expression was induced in hPSCs for 3 days to drive differentiation to CD31+ CD34+ iETV2‐EPCs in a basal medium without growth factors or serum. During Stage 2, iETV2‐EPCs were further differentiated and expanded in serum‐free hECSR medium on collagen IV matrix for two or more days to generate approximately 99% pure CD31+ CD144+ iETV2‐ECs.

We performed RNA sequencing at both iETV2‐EPC and iETV2‐EC stages to benchmark against ECs differentiated from the same hPSC line through mesoderm progenitors. Our analysis indicated that iETV2‐EPC lost the undifferentiated pluripotent stem cell gene expression signature after Stage I, and expressed endothelial markers similarly to meso‐EPCs. After Stage II, iETV2‐ECs demonstrated strong expression of endothelial markers, with a significant correlation in EC marker expression between iETV2‐ECs and meso‐ECs. Flow cytometry and ICC confirmed correct expression and morphology of key endothelial markers in iETV2‐ECs while cord formation, uptake of acetylated LDL and increased adhesion molecule expression upon cytokine treatment confirmed several EC functions of iETV2‐ECs. While cord formation of iETV2‐ECs was not as significant as HUVEC, the observation and mechanism have been reported for other hPSC‐derived ECs (Bezenah et al. 2018). Overall, the endothelial gene expression and endothelial characteristics of iETV2‐ECs generated by this optimized method is comparable to previously published methods for generating hPSC‐derived ECs by either ETV2 overexpression or through mesoderm progenitors (Lian et al. 2014; Ng et al. 2020; Wang et al. 2020). However, the iETV2‐EC differentiation protocol presented in this study, compared to EC differentiation protocols through mesoderm progenitors, is more facile and scalable.

ECs in the brain gain special characteristics to form the blood‐brain barrier compared to peripheral counterparts. Due to its restrictive nature for trafficking of nutrients, waste products and therapeutics between the blood and the CNS, the BBB plays a central role in the regulation and treatment of CNS disease (Rosenberg 2012). hPSC‐derived BBB models possess particular value as they offer robust platforms for studying BBB development (Dao et al. 2024; Gastfriend et al. 2021), disease modeling (Katt et al. 2016; Page et al. 2020; Sun et al. 2020), and drug screening (Li et al. 2019; Ohshima et al. 2019). To explore whether iETV2‐ECs could potentially be adapted for BBB modeling efforts, we leveraged recent research that has highlighted signaling pathways that contribute to BBB development and maintenance and that have been proven to be effective in eliciting BBB induction in hPSC‐derived ECs. Wnt ligands, mainly Wnt7a and Wnt7b, secrete from nearby CNS is essential for BBB development and maintenance (Cho et al. 2017; Cullen et al. 2011; Guérit et al. 2021; Vanhollebeke et al. 2015). Previous research has shown that Wnt activation in meso‐EPCs can induce several BBB properties including GLUT‐1 expression and barrier formation (Gastfriend et al. 2021). Moreover, inhibition of canonical TGFβ signaling and activation of noncanonical TGFβ signaling has been shown to contribute to barriergenesis (Zarkada et al. 2021), and inhibition of TGFβ signaling has been demonstrated to lead to increased claudin‐5 expression and barrier function in meso‐ECs (Roudnicky et al. 2020). In this study, we demonstrated that iETV2‐ECs also respond to these signaling pathways to acquire BBB properties, such as elevated glucose uptake due to increased GLUT1 expression and elevated barrier phenotype due to increased claudin‐5 expression. These findings highlight that iETV2‐ECs, a highly scalable, easy to differentiate cell type can potentially be purposed as an alternative for meso‐ECs for developmental and disease modeling studies at the BBB.

In summary, we present a differentiation strategy to obtain hPSC‐derived endothelial cells through transient ETV2 overexpression in an engineered hPSC lines. We highlighted the ability to achieve 99% CD31+ CD144+ endothelial population in 5 days without the need of delivery of modified RNA or cell sorting. We also demonstrated further patterning iETV2‐EC to ECs exhibiting brain‐like phenotypes representative of the BBB. We anticipate that iETV2‐ECs can serve as an easily obtained, scalable cell source for human endothelial cell research.

## 4 Materials and Methods

### 4.1 Maintenance of hPSCs

Human induced pluripotent stem cells (IMR90‐4 from WiCell WISCi004‐B, IMR90‐4 iETV2) were maintained on Matrigel (Corning 356234) coated plates in mTeSR (Stemcell Technologies 85850). Human ESCs (H9 from WiCell WA09, H9 iETV2) were maintained on Matrigel (Corning 356234) coated plates in mTeSR (Stemcell Technologies 85850). Edited iETV2 hPSCs were maintained with 50 μg/mL hygromycin B (Life Technologies 10687010) to prevent silencing. Quarterly mycoplasma and G‐band karyotyping tests were performed and sterility was monitored daily.

### 4.2 Generation of hPSC iETV2 Cell Lines

A PiggyBac plasmid for doxycycline‐inducible overexpression PB‐TRE‐dCas9‐VPR (Addgene #63800) was obtained. We replaced the dCas9‐VPR construct with a codon‐optimized sequence of ETV2 coding sequence (RefSeq: NM_014209.4). The resulting ~9.1 kb plasmid (PB‐TRE‐ETV2) was transformed into NEB Stable Competent E. coli (New England Biolabs C3040H) and grown in Terrific Broth. Plasmid DNA was purified with the ZymoPURE Plasmid Midiprep Kit (Zymo D4200). IMR90‐4 and H9 hPSCs were reverse‐transfected with a Super PiggyBac Transposase Expression Vector (System Biosciences PB210PA‐1) and PB‐TRE‐ETV2 plasmids with TransIT‐LT1 Transfection Reagent (Mirus Bio 2304) according to the manufacturer's protocol. Four days after transfection, medium was replaced daily with mTeSR medium supplemented with 50 μg/mL hygromycin to select cells with stable integration of PB‐TRE‐ETV2 constructs.

Next, we generate clonal lines. 200 singularized hygromycin‐resistant cells were seeded on a 10 cm Matrigel‐coated cell culture dish (Corning 353003) in mTeSR with CloneR2 (Stemcell Technologies 100‐0691). After 7 days, 12 colonies were picked from the 10 cm dish and transferred to individual wells on a Matrigel‐coated 12‐well plate in mTeSR with CloneR2. For candidate clones, gDNA was extracted using the Monarch Genomic DNA Purification Kit (New England Biolabs T3010S), and PB‐TRE‐ETV2 copy number in each clone was quantified using PiggyBac qPCR Copy Number Kit (System Biosciences PBC100A‐1). Clonal lines were cryopreserved in FreSR‐S medium (Stemcell Technologies 05859).

### 4.3 Differentiation of hPSCs to iETV2‐EPCs and iETV2‐ECS

On Day ‐1, singularized IMR90‐4 iETV2 hPSCs were seeded at different seeding densities ranging from 0.5 million to 4 million cells per well of a six well plate (Corning 3516) coated with Matrigel (Corning 356234) in mTeSR with 10 μM Y‐27632 (Stemcell Technologies 72307). On Day 0 to Day 2 (Stage 1), differentiation medium 1 μg/mL doxycycline (Sigma D9891) was used. Three different medium formulations were tested: mTeSR, LaSR (advanced DMEM/F12, 2.5 mM GlutaMAX Gibco 35050061 and 60 μg/mL ascorbic acid Sigma A92902) as described previously (Bao et al. 2015; Lian et al. 2014), and DMEM/F12 (Life Technologies 11320033). On Day 3, cells containing majorly CD31 + CD34 + iETV2‐EPCs were singularized using Accutase (Innovative Cell Technologies, AT104), and seeded on tissue culture plates coated with collagen IV (Sigma C5533) at 0.5 million cells per cm^2^ in endothelial medium. On Day 4, a medium change was performed. Two different endothelial medium were tested for endothelial cell expansion on and after Day 3 (Stage 2): hECSR (hESFM Gibco 11111044 with 2% B‐27 Gibco 17504044 and 20 ng/mL FGF2 Peprotech 100‐18B) and EGM2 (Lonza CC‐3202). Cells were analyzed on Day 5.

### 4.4 Differentiation of hPSCs to meso‐EPCs and meso‐ECs

Differentiation was performed according to our previously published methods (Bao et al. 2015; Lian et al. 2014). Briefly, on Day ‐3, singularized IMR90‐4 iPSCs were seeded onto 12‐well plates (Corning 3513) coated with Matrigel at a density of 30,000 cells per well in mTeSR. On Days ‐2 and ‐1, medium changes with mTeSR were performed. On Day 0 and Day 1, cells were treated with 6 μM CHIR99021 (Tocris 4423) for 48 h in LaSR medium. From Day 2 to Day 5, cells were treated with LaSR medium with 50 ng/mL VEGF (Peprotech 100‐20). On Day 5, typically 15%–30% of cell populations are CD31+. On Day 5, meso‐EPCs were purified by magnetic sorting of CD31+ cells, using anti‐CD31‐biotin antibody (Miltenyi 130‐110‐667), anti‐biotin microbeads (Miltenyi 130‐097‐046) and a QuadroMACS separator (Miltenyi 130‐091‐051). Magnetic sorting was performed according to the manufacturer's recommendations. Purified CD31+ meso‐EPCs were further differentiated to meso‐ECs in hECSR medium for 2 days before analysis.

### 4.5 RNA Sequencing and Bioinformatics Analysis

ETV2‐EPCs, meso‐EPCs, iETV2‐ECs and meso‐ECs were differentiated as described above. On day of collection, cells were purified using magnetic‐activated cell sorting (MACS) with human CD31 microbead kit (Miltenyi 130‐091‐935) on a QuadroMACS separator (Miltenyi 130‐091‐051). CD31‐enriched cells were then treated with Tri Reagent (Zymo R2050), Total RNA was then purified using Direct‐zol RNA purification kits (Zymo R2051). PolyA mRNA enrichment and sequencing library preparation was performed by Novogene (Sacramento, CA). Sequencing was performed on a NovaSeq. 6000 (Illumina).

We also obtained publicly available transcriptomic data for H9 (GSE239396), HUVEC (GSE220509, GSE256181, GSE221514), human aortic, lymphatic and microvascular ECs (GSE128179) from gene expression omnibus. FASTQ files were aligned to the human genome (hg38) using RNA STAR (Dobin et al. 2013). Gene counts were then quantified using featureCounts (Liao et al. 2014), and then inputted to DESeq. 2 (Love et al. 2014) for differential expression analysis. TPM was calculated from gene counts and presented in heatmaps. The Wald test with Benjamini–Hochberg correction was used to generate adjusted *p*‐values in differential gene expression analysis. Principal component analysis was performed on counts after the DESeq. 2 (Love et al. 2014).

### 4.6 Immunostaining

Cells were fixed with −20°C methanol (Sigma, 67‐56‐1) for 15 min at room temperature. Following three washes in DPBS the cells were blocked in 10% goat serum (Sigma; G9023) for 30 min. Cells were then incubated with primary antibodies at indicated dilution ratios at 4°C overnight (Table 1). Cells were then washed with PBS three times. Cells were then incubated with secondary antibodies (Table 1) and 20 uM Hoechst 33342 (Thermo Scientific; 62249) for 1 h at room temperature. Cells were then washed three times. For immunofluorescence microscopy, we used a Nikon Ti2‐E microscope. For quantification of immunocytochemistry images, background signals from a secondary antibody only control were deducted from all conditions.

**Table 1:** Antibodies used in this study.

Antibody	Source	Dilution ratios
Anti‐OCT3/4 (recombinant human IgG1)	Miltenyi REA622	1:50 (Flow Cytometry)
Anti‐NANOG (recombinant human IgG1)	Miltenyi REA314	1:50 (Flow Cytometry)
Anti‐CD31 (recombinant human IgG1)	Miltenyi REA730	1:50 (Flow Cytometry)
Anti‐CD144 (recombinant human IgG1)	Miltenyi REA199	1:50 (Flow Cytometry)
Anti‐ZO‐1 (mouse monoclonal IgG1, Clone 1A12)	Invitrogen 33‐9100	1:100 (ICC)
Anti‐CD31 (mouse monoclonal IgG2a, Clone 390)	Invitrogen 14‐0311‐82	1:100 (ICC)
Anti‐CD144 (mouse monoclonal IgG2a, clone BV9)	Santa Cruz Biotechnology sc‐52751	1:100 (ICC)
Anti‐GLUT1 (rabbit IgG, clone SA0377)	Invitrogen MA5‐31960	1:100 (ICC)
Anti‐claudin‐5 (mouse monoclonal IgG1, clone 4C3C2)	Invitrogen 35‐2500	1:100 (ICC)
Alexa Fluor 488 goat anti‐mouse IgG (goat polyclonal)	Invitrogen A‐28175	1:200 (ICC), 1:200 (Flow Cytometry)
Alexa Fluor 647 goat anti‐rabbit IgG (goat polyclonal)	Invitrogen A‐21245	1:200 (ICC), 1:200 (Flow Cytometry)

### 4.7 Flow Cytometry

Cells were singularized with Accutase, pelleted, and resuspended in flow buffer, composed of DPBS (Gibco 14190144) with 0.5% bovine serum albumin (Sigma A2153) and 2 mM EDTA (Sigma; 03620) with pre‐conjugated antibody at 4°C for 15 min (Table 1). Cells were then pelleted and washed three times with flow buffer. Data were collected on a BD Accuri C6 Plus flow cytometer and analyzed using FlowJo.

### 4.8 Reverse Transcription Real‐Time PCR Analysis

RNA was extracted using Direct‐zol RNA Miniprep kit (Zymo; R2050). Reverse transcription was performed to obtain cDNA using GoScript Reverse Transcriptase with Oligo(dT) kit (Promega; A2791). Real‐time gene expression analysis was conducted using 25 μL reactions containing SYBR Green PCR Master Mix (Life Technologies; 4309155) along with primers specific for gene of interests (Table 2). PCR was run according to manufacturer protocols on Agilent AriaMX Real‐Time PCR system.

**Table 2:** Primer sequences for RT‐qPCR used in this study.

Gene	Forward Primer (5'‐3')	Reverse Primer (5'‐3')
*CLDN5*	TGACCTTCTCCTGCCACTA	AAGCGAAATCCTCAGTCTGAC
*SLC2A1*	GTGCCATACTCATGACCATCG	GGCCACAAAGCCAAAGATG
*HPRT1*	TTGTTGTAGGATATGCCCTTGA	GCGATGTCAATAGGACTCCAG

### 4.9 Cord Formation Assay

Differentiated iETV2‐ECs and meso‐ECs were seeded at 80,000 cells per cm^2^, while HUVECs were seeded at 40,000 cells per cm^2^ on 24‐well plates coated with 50 µL per cm^2^ Geltrex (Gibco A1413201) in Medium 200PRF (Gibco M200PRF500) supplemented with LSGS (Gibco S00310), 2% FBS (Gibco A3160501) and 3 ng/mL bFGF (Gibco 13256‐029). Time‐course brightfield images were taken using a Nikon Ti2‐E microscope every 15 min for 3 h with cells incubating on an on‐microscope temperature, humidity and CO_2_‐controlled chamber.

### 4.10 Glucose Uptake Assay

Glucose uptake assay was performed using Glucose Uptake‐Glo kit (Promega J1341) according to the manufacturer's recommendation. Briefly, differentiated Day 5 iETV2‐ECs treated with or without CHIR99021 in stage 2 medium were seeded at 40,000 cells per well in 96‐well plates coated with 50 μL of 100 μg/mL collagen IV (Sigma C5533). Cells were incubated with 2‐deoxyglucose for 10 min during which 2‐deoxyglucose is converted to 2‐deoxyglucose‐6‐phosphate inside cells. Stop buffer and neutralization buffer were then added to stop reaction and lyse the cells. 2‐deoxyglucose‐6‐phosphate detection reagent was then added, which reports 2‐deoxyglucose‐6‐phosphate concentration as luciferin, detected on a Tecan Infinite 200 PRO plate reader.

### 4.11 Transendothelial Electrical Resistance

Transwell inserts (Corning 3460) were coated with 200 µL of 100 μg/mL collagen IV in water overnight at 37°C. Day 5 iETV2‐ECs treated with DMSO, 4 μM CHIR99021, or 4 μM CHIR99021 and 10 uM RepSox were seeded on Transwell inserts at 100,000 cells/cm^2^ in hECSR medium supplemented with small molecules. Beginning 2 days after seeding, TEER was measured daily for 6 days using an EVOM2 voltohmmeter with STX2 chopstick electrodes (World Precision Instruments). Medium was replaced every other day. TEER values were corrected by subtracting the reading from a collagen IV‐coated Transwell insert without cells and multiplying by the surface area of 1.1 cm^2^.

### 4.12 Statistics

Biological replicates in this manuscript refer to individual wells of cultured cells that underwent identical experimental treatments. The authors of the study ensured that all key experiments were repeated using one iPSC line and one hESC line. Detailed replication strategy is stated in figure legends. To compare means of two experimental groups, Student's *t*‐test was used. For experiments with three or more experimental groups, one‐way analysis of variance (ANOVA) was used for comparison of means. Following ANOVA, Dunnett's post hoc test was used for the comparison of multiple treatments to a single control, or Tukey's honest significant difference test was used for multiple pairwise comparisons. Statistical tests were performed in GraphPad Prism. Descriptions of the statistical tests used are provided in figure legends.

## Author Contributions

**Yunfeng Ding:** conceptualization, experiments, writing – original draft preparation. **Soniya Tamhankar:** experiments, writing – original draft preparation. **Feifan Du, Tessa Christopherson, Nate Schlueter, and Jenna R. Cohen:** experiments**. Eric V. Shusta:** conceptualization, writing – review and editing, supervision. **Sean P. Palecek:** conceptualization, writing – review and editing, supervision.

## Conflicts of Interest

The authors have multiple issued patents and patent applications in the field of BBB modeling.

## Supporting information

Supporting information.

## Data Availability

All plamids generated by this paper will be available at https://www.addgene.org/Sean_Palecek/. High‐throughput sequencing data obtained in this study have been submitted to GEO and are available under the accession number GEO: GSE271101. This paper does not report original code. Any additional information required to reanalyze the data reported in this paper is available from the corresponding authors upon request. Further information and requests for resources and reagents should be directed to and will be fulfilled by the corresponding authors Sean P. Palecek (sppalecek@wisc.edu; lead contact) and Eric V. Shusta (eshusta@wisc.edu).
