# Supplementary material for: ETV2 Overexpression Promotes Efficient Differentiation of Pluripotent Stem Cells to Endothelial Cells
Source: Biotechnol Bioeng. 2025 Mar 25;122(7):1914–28. doi: 10.1002/bit.28979 (PMC12152468; doi:10.1002/bit.28979)
Supplement: Supplementary file 1 — Supporting information. [file BIT-122-1914-s001.pdf]

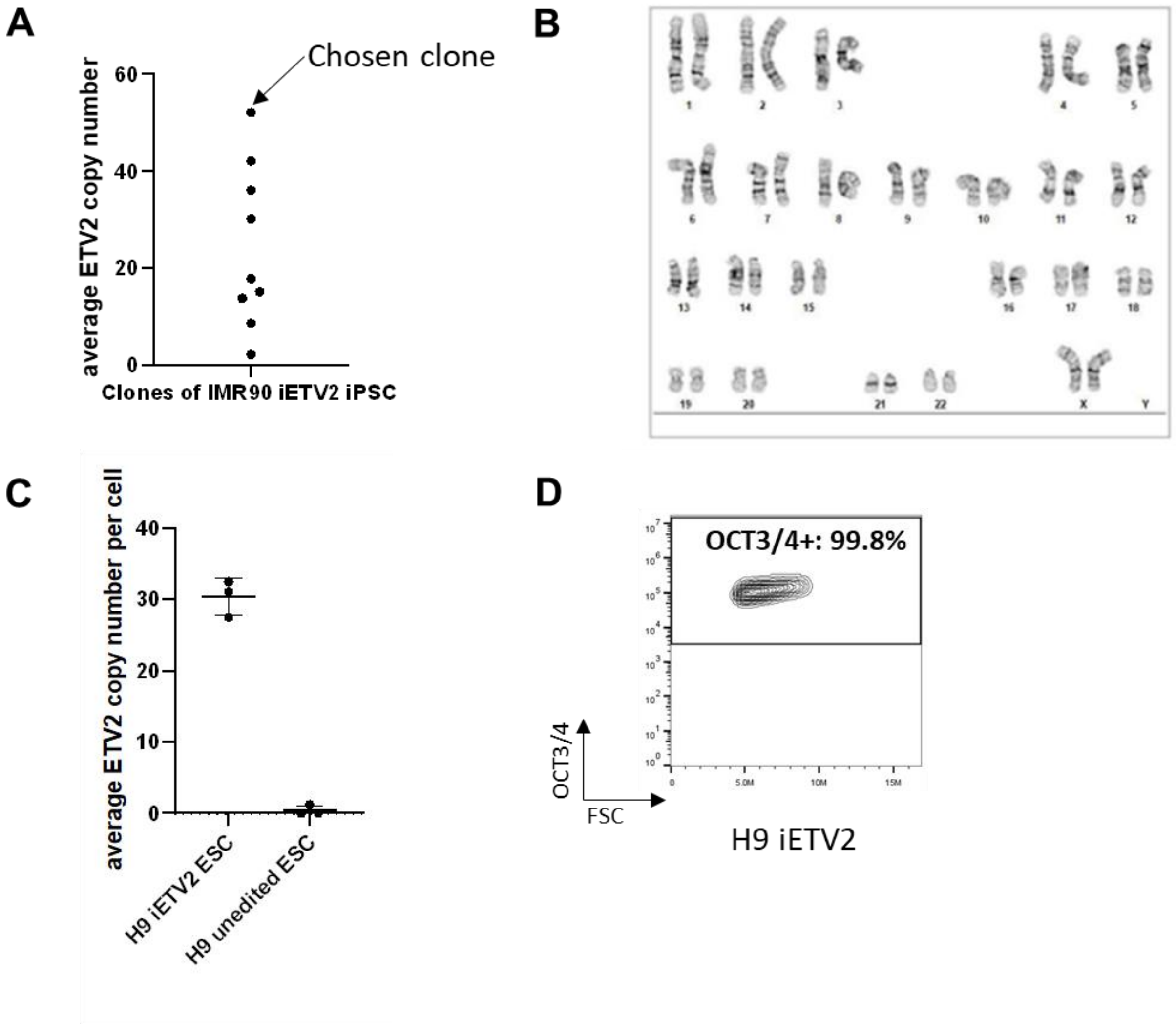


**Figure S1. Characterization of iETV2 hPSC lines**

(A) qPCR on genomic DNA of clonally selected IMR90-4 iETV2 iPSCs clones. Each point represents an individual isolated clone. The clone with the highest copy number (52 copies) was selected for detailed characterization.

(B) G-band karyotyping of IMR90-4 iETV2 iPSCs with an average copy number of 52 indicated no chromosomal abnormalities of the edited line.

(C) qPCR of genomic DNA of clonally-selected edited and unedited H9 ESCs indicated an average of 30 genomic copies of iETV2 constructs in the edited clone. N=3 independent genomic DNA isolations from each line.

(D) Flow cytometry of H9 iETV2 ESCs indicated that H9 iETV2 ECSs continue to express pluripotency marker OCT3/4. Isotype control is the same as in Figure 1C.

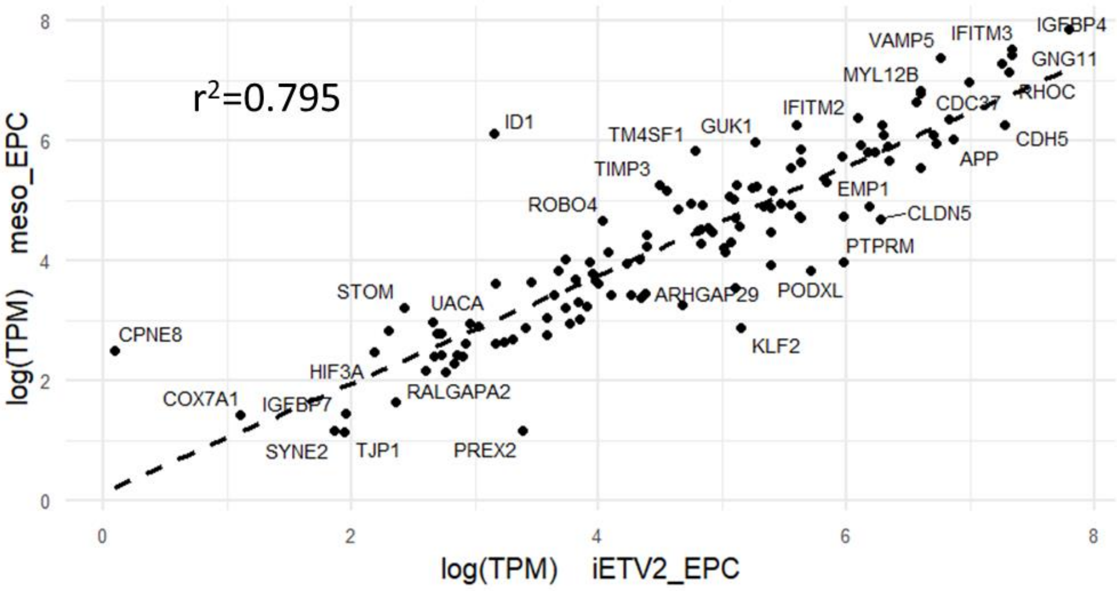


**Figure S2. iETV2-EPCs and meso-EPCs have similar expression of endothelial genes.**

(A) Scatter plot showing the log expression of 125 pan-endothelial genes in iETV2-EPCs and meso-EPCs. Linear regression of log expression was performed and revealed high correlation of endothelial gene expression between the two cell types.

**A**

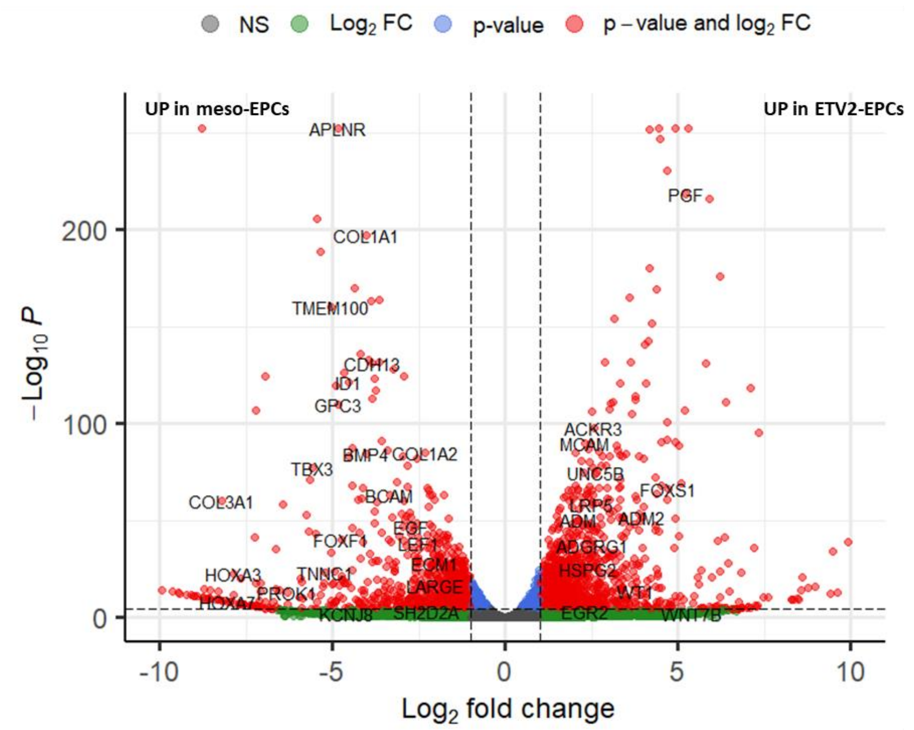


**B**

| Upregulated GO Biological Process in ETV2-EPCs | Fold Enrichment | FDR |
|---|---|---|
| vagus nerve development (GO:0021564) | 27.74 | 4.91E-03 |
| negative regulation of dopamine secretion (GO:0033602) | 20.8 | 1.47E-02 |
| atrial cardiac muscle cell membrane repolarization (GO:0099624) | 16.64 | 2.73E-02 |
| postsynaptic density assembly (GO:0097107) | 13.87 | 3.10E-04 |
| positive regulation of natural killer cell differentiation (GO:0032825) | 13.87 | 9.46E-03 |

**C**

| Upregulated GO Biological Process in meso-EPCs | Fold Enrichment | FDR |
|---|---|---|
| specification of animal organ position (GO:0010159) | 33.03 | 1.96E-03 |
| fibroblast growth factor receptor apoptotic signaling pathway (GO:1902178) | 33.03 | 2.96E-02 |
| negative regulation of phosphatidylcholine metabolic process (GO:0150174) | 33.03 | 2.94E-02 |
| semicircular canal formation (GO:0060876) | 33.03 | 2.94E-02 |
| cellular response to interleukin-18 (GO:0071351) | 16.52 | 1.93E-02 |

**Figure S3: Differential gene expression analysis between ETV2-EPCs and meso-EPCs.**

(A) Volcano plot displaying differential gene expression analysis results. X-axis is $\log_2$ fold change of gene expression. Y-axis is the $-\log_{10}$ of the adjusted p value of DESeq2 analysis. $\text{Log}_2$ fold change larger than 1 was chosen as the fold change cut off, adjusted p value of 0.05 was chosen as the p-value cut off. Selected differentially expressed genes that are related to blood vessel development are labeled.

(B) Top 5 gene ontology (GO) terms upregulated in ETV2-EPCs.

(C) Top 5 gene ontology (GO) terms upregulated in meso-EPCs.

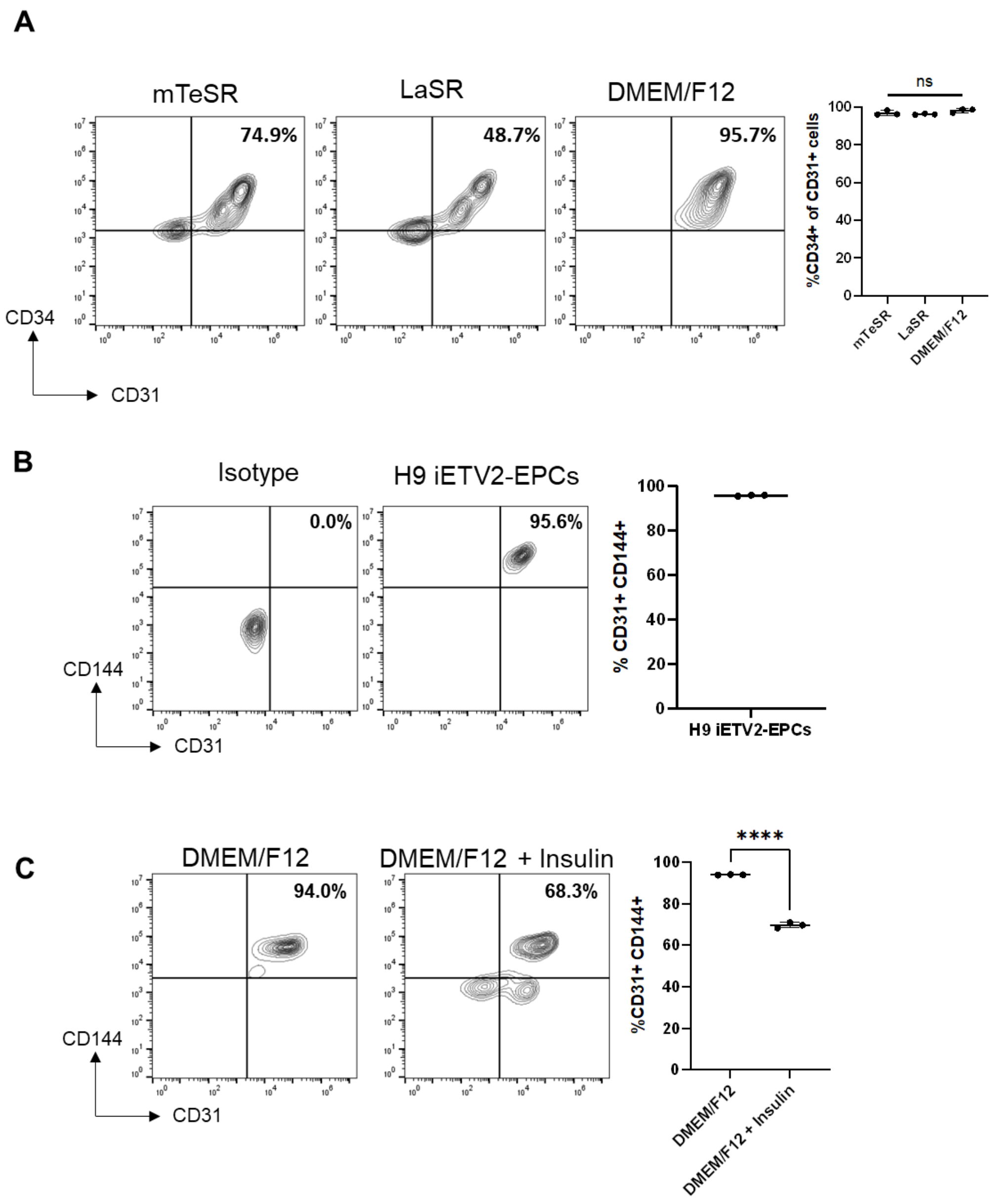


**Figure S4. DMEM/F12 and optimal seeding density during Stage 1 yields Day 3 cells with high purity CD31+ CD144+ populations.**

(A) Flow cytometry analysis of CD34 and CD31 expression of day 3 IMR90-4 iETV2-EPCs differentiated in mTeSR, LaSR, or DMEM/F12 during Stage 1. Seeding density was 1 million cells/well in a six-well plate. N=3 independently differentiated biological replicates were quantified. ns: $p>0.05$ in one-way ANOVA followed by Tukey's test.

(B) Flow cytometry analysis of CD144 and CD31 expression in day 3 H9 iETV2-EPCs differentiated in DMEM/F12 during Stage 1. Seeding density was 2 million cells / well in a six-well plate. N=3 independently differentiated biological replicates.

(C) Flow cytometry analysis of CD144 and CD31 expression of day 3 IMR90-4 iETV2-EPCs differentiated in DMEM/F12 or DMEM/F12 supplemented with 10 ug/mL insulin during Stage 1. Seeding density was 1 million cells/well in a six-well plate. N=3 independently differentiated biological replicates were quantified. ****: $p<0.0001$ in one-way ANOVA followed by Tukey's test.

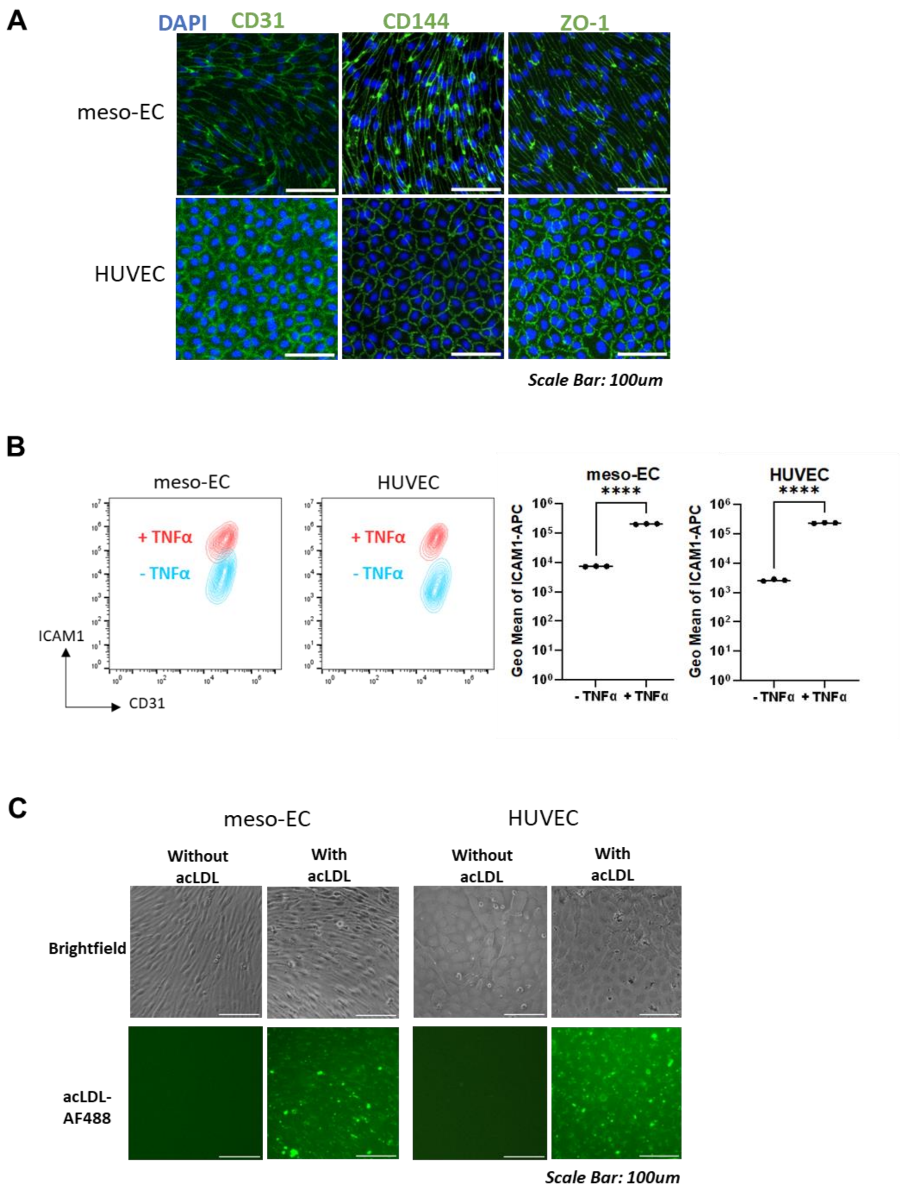


**Figure S5. iETV2-ECs are similar to meso-ECs and HUVECs in endothelial protein expression and function.**

(A) Immunofluorescent images showing CD31, CD144 and ZO-1 expression in meso-ECs differentiated from the IMR90-4 iPSC line and HUVECs, cultured in hECSR medium. Scale bars are 100 μm

(B) Flow cytometry analysis of ICAM1 expression after 24 hours of incubation with or without TNFα in meso-ECs differentiated from the IMR90-4 iPSC line using and HUVECs. Red: with TNFα. Blue: Without TNFα. Geometric mean of ICAM1 expression was also quantified. N=3 independently differentiated biological replicates were quantified. ****: p<0.0001 in Student's t-test.

(C) Brightfield and immunofluorescence images of meso-ECs differentiated from the IMR90-4 iETV2 line, and HUVECs. Images were taken with or without 24 hr incubation with Alexa Fluor 488-labeled acetylated LDL (acLDL-AF488). Scale bars are 100 μm.

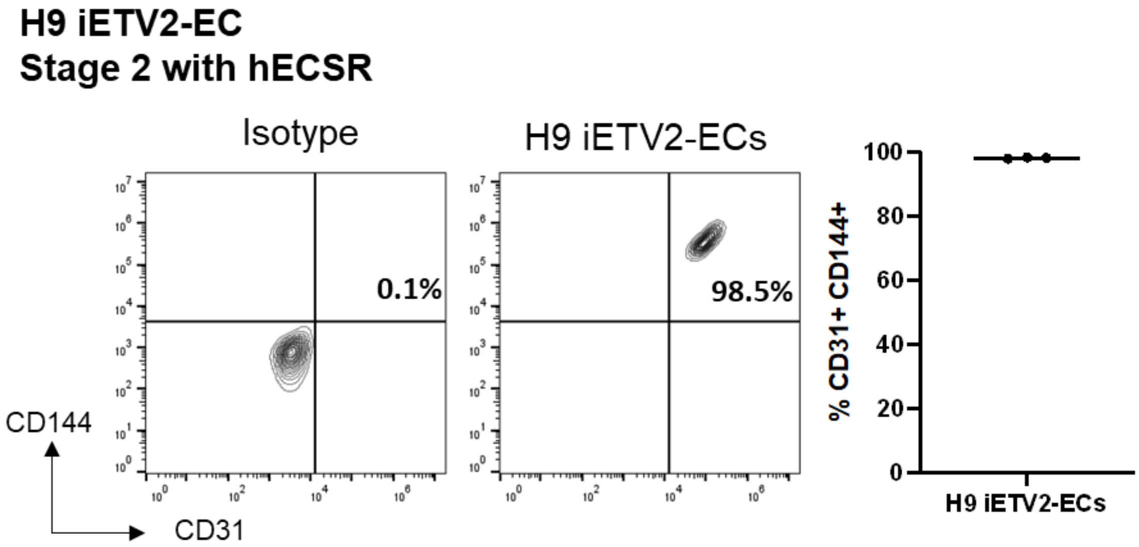


**Figure S6. Flow cytometry demonstrates that using hECSR during Stage 2 expansion yielded Day 5 cells with high purities of CD31+ CD144+ populations.**
Flow cytometry analysis on the expression of CD144 and CD31 on day 5 H9 iETV2-ECs expanded in hECSR. N=3 independently differentiated biological replicates.

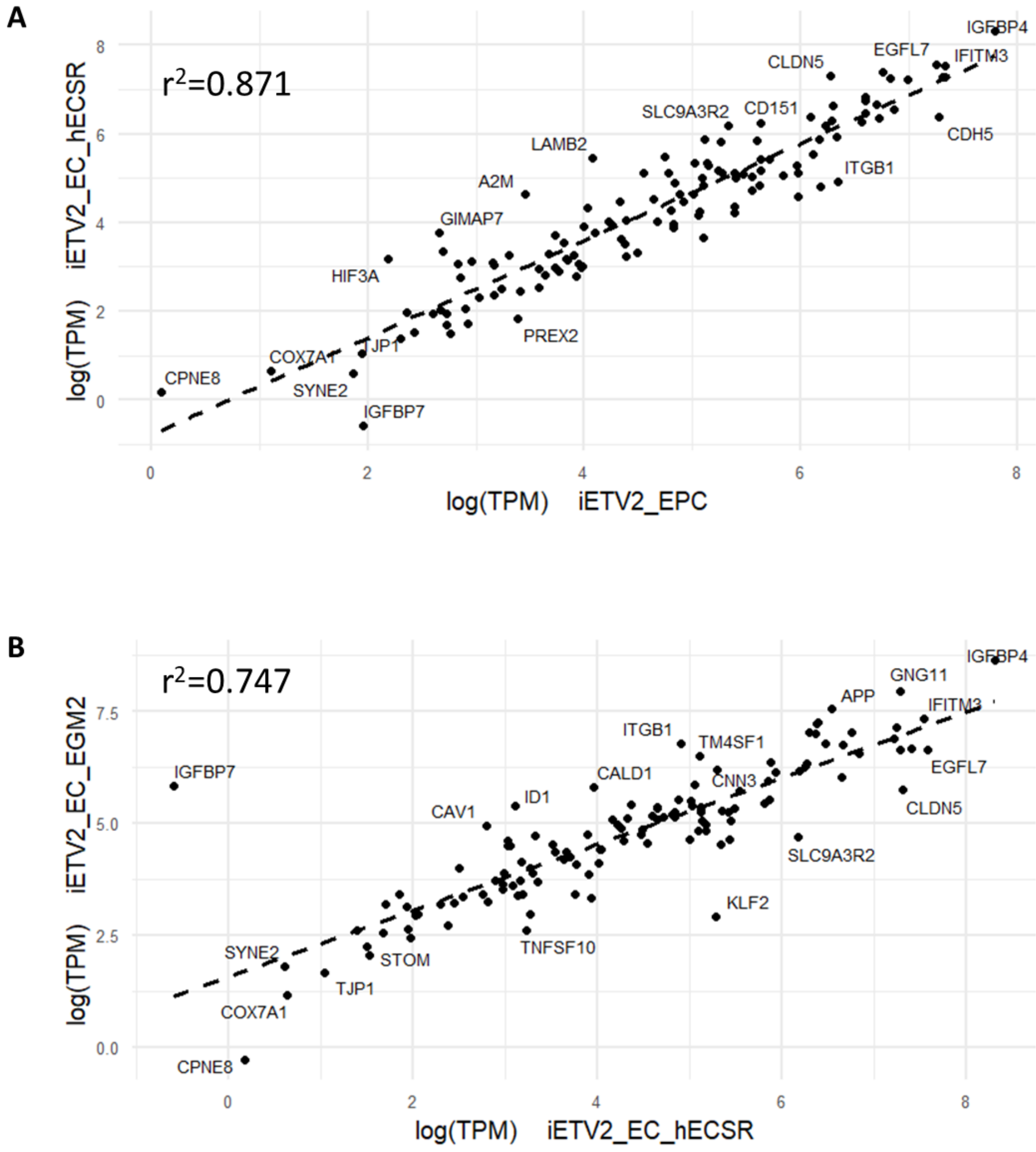


**Figure S7. Choice of Stage 2 expansion media and expansion culture does not significantly affect endothelial gene expression.**

(A) Scatter plot showing the log expression of 125 pan-endothelial genes in iETV2-EPCs and iETV2-ECs expanded in hECSR. Linear regression of log expression was performed and revealed high correlation of endothelial gene expression between the two cell populations.

(B) Scatter plot showing the log expression of 125 pan-endothelial genes in iETV2-ECs expanded in hECSR and iETV2-ECs expanded in EGM2. Linear regression of log expression was performed and revealed high correlation of endothelial gene expression between the two cell populations.

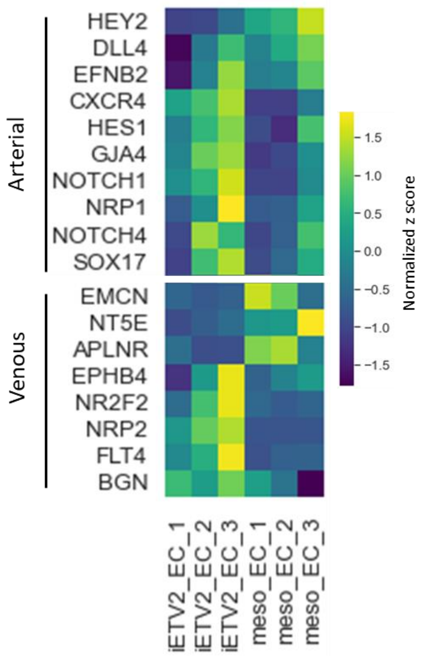


**Figure S8: Expression of arterial and venous genes in iETV2-ECs and meso-ECs.** Heatmap indicating the expression of a panel of arterial EC markers (*HEY2, DLL4, EFNB2, CXCR4, HES1, GJA4, NOTCH1, NRP1, NOTCH4, SOX17)* and a panel of venous EC markers (*EMCN, NT5E, APLNR, EPHB4, NR2F2, NRP2, FLT4, BGN*) in iETV2-ECs and meso-ECs. Lists of arterial and venous genes are from Pan et al., 2024.

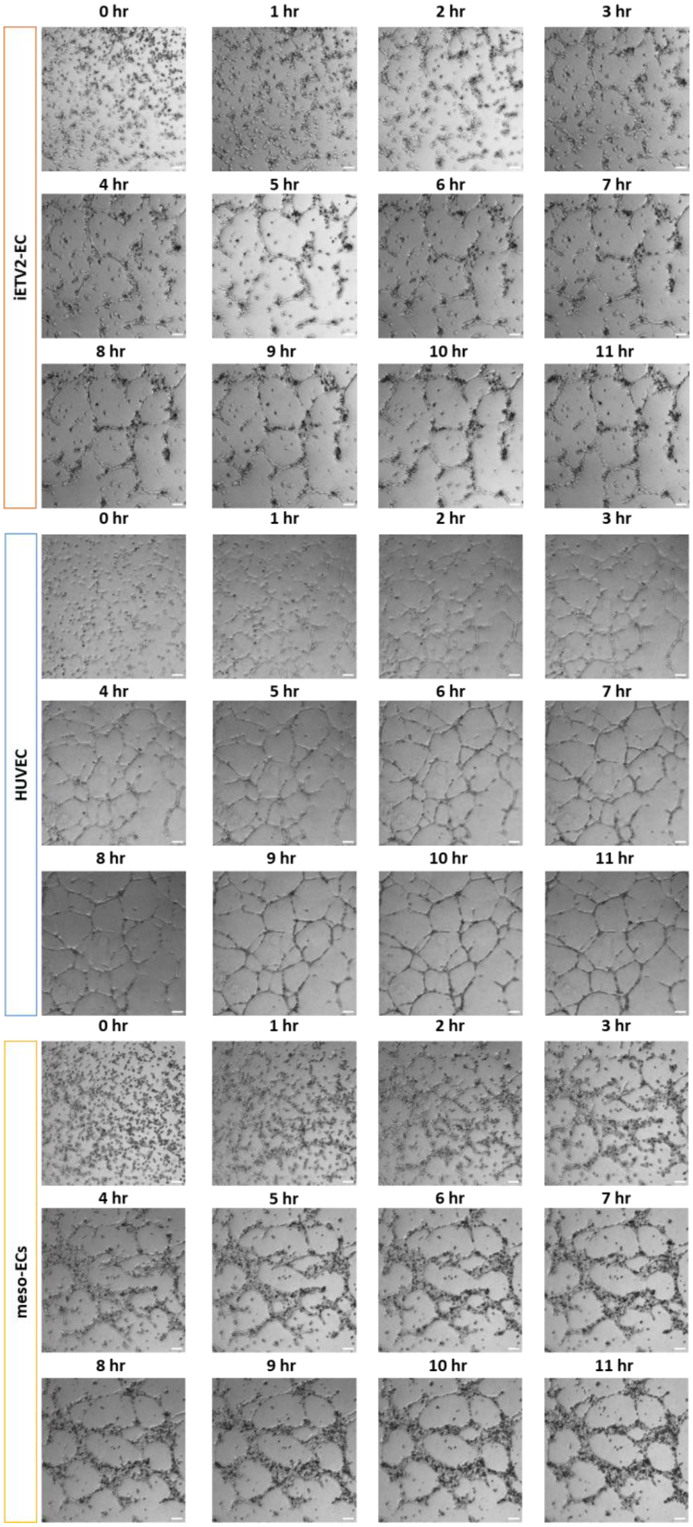


**Figure S9. Time-course images of cord formation assay performed on iETV2-ECs, meso-ECs and HUVEC revealed angiogenesis potential of iETV2-ECs.**

Cord formation assay for iETV2-ECs, HUVECs and meso-ECs from 0 hr to 11 hr after medium change to angiogenic, low serum growth supplement. A starting density of 40,000 cells per cm2 were used for HUVECs, while a starting density of 80,000 cells per cm2 were used for iETV2-ECs and meso-ECs.

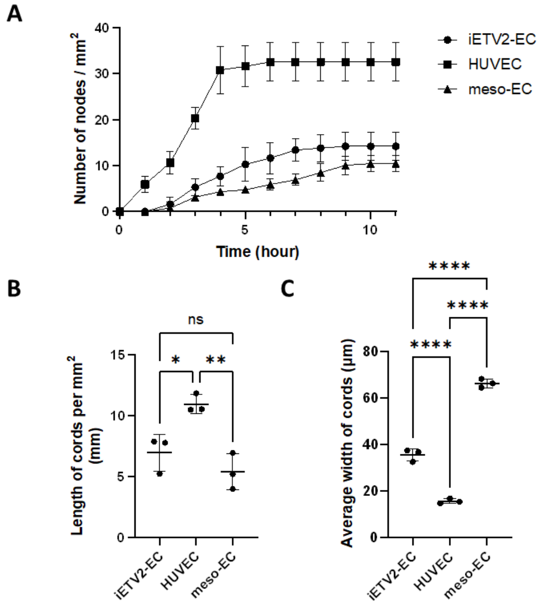


**Figure S10: Quantification of cord formation capabilities of iETV2-ECs, HUVECs and meso-ECs.**

(A) Number of nodes per $mm^2$ from 0 hr to 11 hr after medium change to angiogenic, low serum growth supplement. A starting density of 40,000 cells per $cm^2$ were used for HUVECs, while a starting density of 80,000 cells per $cm^2$ were used for iETV2-ECs and meso-ECs. A node is defined as a point where two or more branches of cords intersect. N=3 independently treated biological replicates were counted.

(B) Total length of cords per $mm^2$ 11 hr after medium change to angiogenic, low serum growth supplement. All cords in a replicate were measured in length and the length values are summed. The summed total lengths of N=3 independently treated biological replicates were quantified. *: $p<0.05$, **:$p<0.01$, ns: not significant in One-way ANOVA followed by Tukey's test.

(C) Average width of cords 11 hr after medium change to angiogenic, low serum growth supplement. All cords in a replicate were measured in width and the width values are averaged. The averaged cord width of N=3 independently treated biological replicates were quantified. ****: $p<0.0001$ in One-way ANOVA followed by Tukey's test.

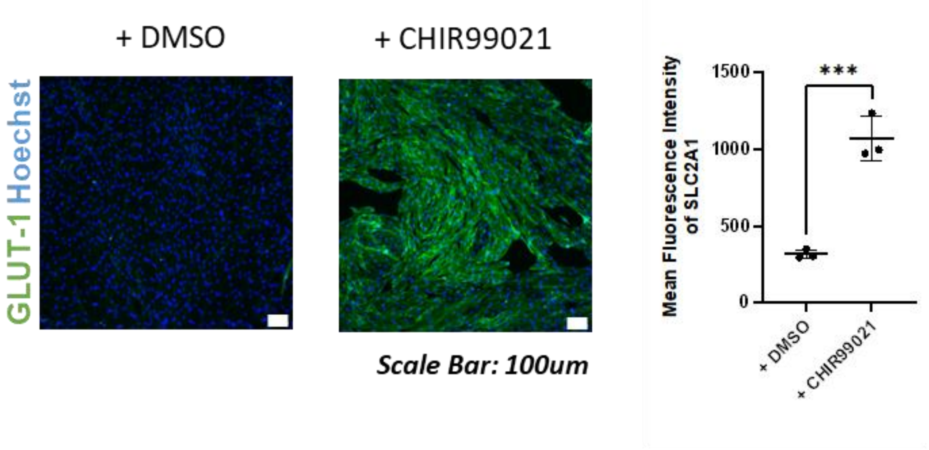


**Figure S11: Expression of GLUT-1 in meso-ECs upon CHIR99021 treatment.**
Immunofluorescent images showing GLUT-1 expression in IMR90-4 meso-ECs treated with Wnt agonist CHIR99021 or DMSO control. Scale bars are 100 µm. Quantification of mean GLUT-1 fluorescence intensity in immunofluorescent images was performed. N=3 independently treated biological replicates were quantified. ***: $p<0.001$ in Student's t-test.
